# Supplementary material for: Intrinsic Disorder of the BAF Complex: Roles in Chromatin Remodeling and Disease Development
Source: Int J Mol Sci. 2019 Oct 23;20(21):5260. doi: 10.3390/ijms20215260 (PMC6862534; doi:10.3390/ijms20215260)

Supplementary Materials

Intrinsic Disorder of BAF Complex: Roles in Chromatin Remodeling and Disease Development

Nashwa El Hadidy [1] and Vladimir N. Uversky [1,2,*]

[1] Department of Molecular Medicine, Morsani College of Medicine, University of South Florida, 12901 Bruce B. Downs Blvd. MDC07, Tampa, Florida, USA; E-Mail: nashwa@health.usf.edu (N.E.H.); vuversky@health.usf.edu (V.N.U.)

[2] Laboratory of New Methods in Biology, Institute for Biological Instrumentation, Russian Academy of Sciences, Pushchino142290, Moscow Region, Russia

* Author to whom correspondence should be addressed; E-Mail: vuversky@healh.usf.edu; Tel.: 1-813-974-5816; Fax: 1-813-974-7357.

BRG1/SMARCA4 (UniProt ID: P51532; PPID=61.3%; 1,647 residues)

```
>sp|P51532|SMCA4_HUMAN Transcription activator BRG1 OS=Homo sapiens OX=9606
GN=SMARCA4 PE=1 SV=2
MSTPDPPLGGTPRPGPSPGPGPSPGAMLGPSPGPSPGSAHSMMGPSPGPPSAGHPIPTQGPGGYPQDNMHQMHKPME
SMHEKGMSDDPRYNQMKGMGMRSGGHAGMGPPPSPMDQHSQGYPSPLGGSEHASSPVPASGPSSGPQMSSGPGGAPL
DGADPQALGQQNRGPTPFNQNQLHQLRAQIMAYKMLARGQPLPDHLQMAVQGKRPMPGMQQQMPTLPPPSVSATGPG
PGPGPGPGPGPGPAPPNYSRPHGMGGPNMPPPGPSGVPPGMPGQPPGGPPKPWPEGPMANAAAPTSTPQKLIPPQPT
GRPSPAPPAVPPAASPVMPPQTQSPGQPAQPAPMVPLHQKQSRITPIQKPRGLDPVEILQEREYRLQARIAHRIQEL
ENLPGSLAGDLRTKATIELKALRLLNFQRQLRQEVVVCMRRDTALETALNAKAYKRSKRQSLREARITEKLEKQQKI
EQERKRRQKHQEYLNSILQHAKDFKEYHRSVTGKIQKLTKAVATYHANTEREQKKENERIEKERMRRLMAEDEEGYR
KLIDQKKDKRLAYLLQQTDEYVANLTELVRQHKAAQVAKEKKKKKKKKKAENAEGQTPAIGPDGEPLDETSQMSDLP
VKVIHVESGKILTGTDAPKAGQLEAWLEMNPGYEVAPRSDSEESGSEEEEEEEEEEQPQAAQPPTLPVEEKKKIPDP
DSDDVSEVDARHIIENAKQDVDDEYGVSQALARGLQSYYAVAHAVTERVDKQSALMVNGVLKQYQIKGLEWLVSLYN
NNLNGILADEMGLGKTIQTIALITYLMEHKRINGPFLIIVPLSTLSNWAYEFDKWAPSVVKVSYKGSPAARRAFVPQ
LRSGKFNVLLTTYEYIIKDKHILAKIRWKYMIVDEGHRMKNHHCKLTQVLNTHYVAPRRLLLTGTPLQNKLPELWAL
LNFLLPTIFKSCSTFEQWFNAPFAMTGEKVDLNEEETILIIRRLHKVLRPFLLRRLKKEVEAQLPEKVEYVIKCDMS
ALQRVLYRHMQAKGVLLTDGSEKDKKGKGGTKTLMNTIMQLRKICNHPYMFQHIEESFSEHLGFTGGIVQGLDLYRA
SGKFELLDRILPKLRATNHKVLLFCQMTSLMTIMEDYFAYRGFKYLRLDGTTKAEDRGMLLKTFNEPGSEYFIFLLS
TRAGGLGLNLQSADTVIIFDSDWNPHQDLQAQDRAHRIGQQNEVRVLRLCTVNSVEEKILAAAKYKLNVDQKVIQAG
MFDQKSSSHERRAFLQAILEHEEQDESRHCSTGSGSASFAHTAPPPAGVNPDLEEPPLKEEDEVPDDETVNQMIARH
EEEFDLFMRMDLDRRREEARNPKRKPRLMEEDELPSWIIKDDAEVERLTCEEEEEKMFGRGSRHRKEVDYSDSLTEK
QWLKAIEEGTLEEIEEEVRQKKSSRKRKRDSDAGSSTPTTSTRSRDKDDESKKQKKRGRPPAEKLSPNPPNLTKKMK
KIVDAVIKYKDSSSGRQLSEVFIQLPSRKELPEYYELIRKPVDFKKIKERIRNHKYRSLNDLEKDVMLLCQNAQTFN
LEGSLIYEDSIVLQSVFTSVRQKIEKEDDSEGEESEEEEEGEEEGSESESRSVKVKIKLGRKEKAQDRLKGGRRRPS
RGSRAKPVVSDDDSEEEQEEDRSGSGSEED
```

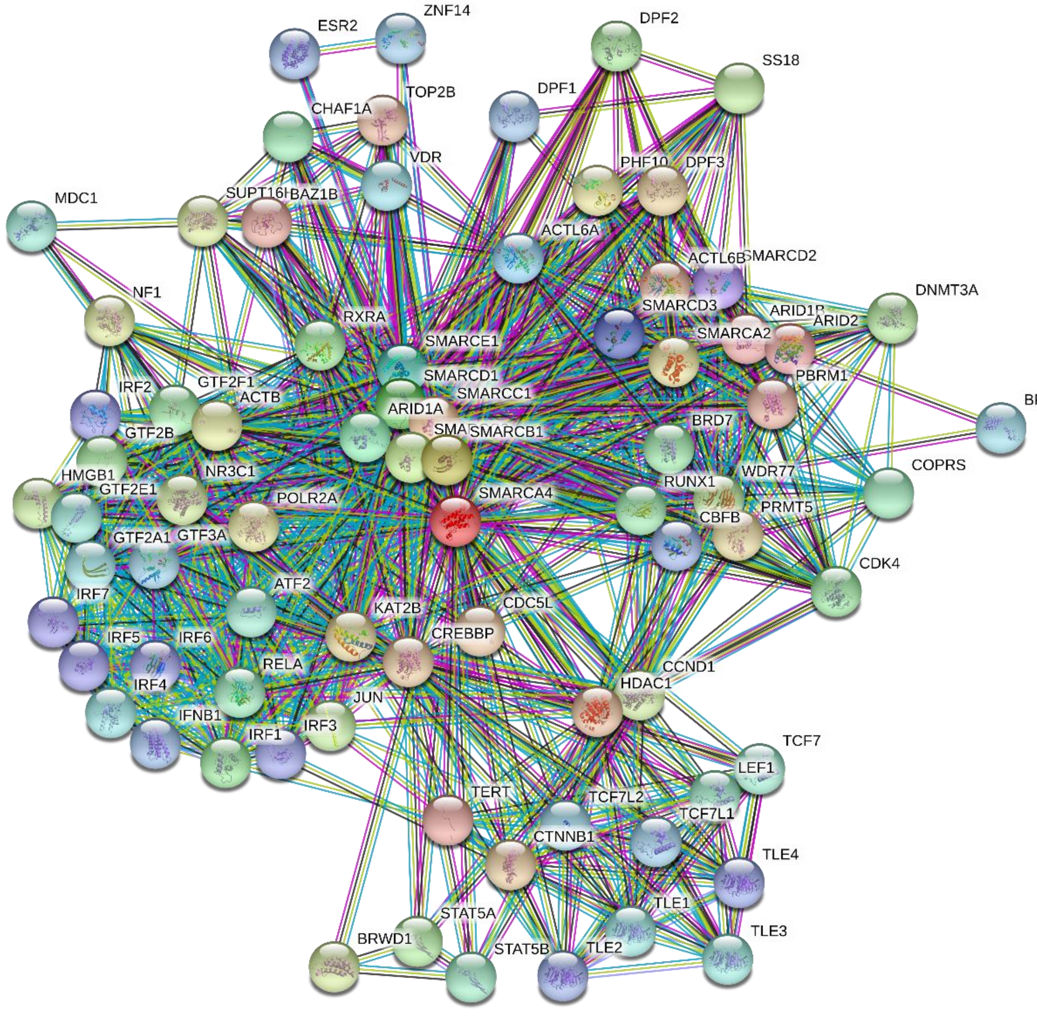

BAF250A/ARID1A (UniProt ID: O14497; PPID=75.8%; 2,285 residues)

```
>sp|O14497|ARI1A_HUMAN AT-rich interactive domain-containing protein 1A
OS=Homo sapiens OX=9606 GN=ARID1A PE=1 SV=3
MAAQVAPAAASSLGNPPPPPPSELKKAEQQQREEAGGEAAAAAAAERGEMKAAAGQESEGPAVGPPQPLGKELQDGA
ESNGGGGGGGAGSGGGPGAEPDLKNSNGNAGPRPALNNNLTEPPGGGGGGSSDGVGAPPHSAAAALPPPAYGFGQPY
GRSPSAVAAAAAAVFHQQHGGQQSPGLAALQSGGGGGLEPYAGPQQNSHDHGFPNHQYNSYYPNRSAYPPPAPAYAL
SSPRGGTPGSGAAAAAGSKPPPSSSASASSSSSSFAQQRFGAMGGGGPSAAGGGTPQPTATPTLNQLLTSPSSARGY
QGYPGGDYSGGPQDGGAGKGPADMASQCWGAAAAAAAAAAASGGAQQRSHHAPMSPGSSGGGGQPLARTPQPSSPMD
QMGKMRPQPYGGTNPYSQQQGPPSGPQQGHGYPGQPYGSQTPQRYPMTMQGRAQSAMGGLSYTQQIPPYGQQGPSGY
GQQGQTPYYNQQSPHPQQQQPPYSQQPPSQTPHAQPSYQQQPQSQPPQLQSSQPPYSQQPSQPPHQQSPAPYPSQQS
TTQQHPQSQPPYSQPQAQSPYQQQQPQQPAPSTLSQQAAYPQPQSQQSQQTAYSQQRFPPPQELSQDSFGSQASSAP
SMTSSKGGQEDMNLSLQSRPSSLPDLSGSIDDLPMGTEGALSPGVSTSGISSSQGEQSNPAQSPFSPHTSPHLPGIR
GPSPSPVGSPASVAQSRSGPLSPAAVPGNQMPPRPPSGQSDSIMHPSMNQSSIAQDRGYMQRNPQMPQYSSPQPGSA
LSPRQPSGGQIHTGMGSYQQNSMGSYGPQGGQYGPQGGYPRQPNYNALPNANYPSAGMAGGINPMGAGGQMHGQPGI
PPYGTLPPGRMSHASMGNRPYGPNMANMPPQVGSGMCPPPGGMNRKTQETAVAMHVAANSIQNRPPGYPNMNQGGMM
GTGPPYGQGINSMAGMINPQGPPYSMGGTMANNSAGMAASPEMMGLGDVKLTPATKMNNKADGTPKTESKSKKSSSS
TTTNEKITKLYELGGEPERKMWVDRYLAFTEEKAMGMTNLPAVGRKPLDLYRLYVSVKEIGGLTQVNKNKKWRELAT
NLNVGTSSSAASSLKKQYIQCLYAFECKIERGEDPPPDIFAAADSKKSQPKIQPPSPAGSGSMQGPQTPQSTSSSMA
EGGDLKPPTPASTPHSQIPPLPGMSRSNSVGIQDAFNDGSDSTFQKRNSMTPNPGYQPSMNTSDMMGRMSYEPNKDP
YGSMRKAPGSDPFMSSGQGPNGGMGDPYSRAAGPGLGNVAMGPRQHYPYGGPYDRVRTEPGIGPEGNMSTGAPQPNL
MPSNPDSGMYSPSRYPPQQQQQQQQRHDSYGNQFSTQGTPSGSPFPSQQTTMYQQQQQNYKRPMDGTYGPPAKRHEG
EMYSVPYSTGQGQPQQQQLPPAQPQPASQQQAAQPSPQQDVYNQYGNAYPATATAATERRPAGGPQNQFPFQFGRDR
VSAPPGTNAQQNMPPQMMGGPIQASAEVAQQGTMWQGRNDMTYNYANRQSTGSAPQGPAYHGVNRTDEMLHTDQRAN
HEGSWPSHGTRQPPYGPSAPVPPMTRPPPSNYQPPPSMQNHIPQVSSPAPLPRPMENRTSPSKSPFLHSGMKMQKAG
PPVPASHIAPAPVQPPMIRRDITFPPGSVEATQPVLKQRRRLTMKDIGTPEAWRVMMSLKSGLLAESTWALDTINIL
LYDDNSIMTFNLSQLPGLLELLVEYFRRCLIEIFGILKEYEVGDPGQRTLLDPGRFSKVSSPAPMEGGEEEEELLGP
KLEEEEEEEVVENDEEIAFSGKDKPASENSEEKLISKFDKLPVKIVQKNDPFVVDCSDKLGRVQEFDSGLLHWRIGG
GDTTEHIQTHFESKTELLPSRPHAPCPPAPRKHVTTAEGTPGTTDQEGPPPDGPPEKRITATMDDMLSTRSSTLTED
GAKSSEAIKESSKFPFGISPAQSHRNIKILEDEPHSKDETPLCTLLDWQDSLAKRCVCVSNTIRSLSFVPGNDFEMS
KHPGLLLILGKLILLHHKHPERKQAPLTYEKEEEQDQGVSCNKVEWWWDCLEMLRENTLVTLANISGQLDLSPYPES
ICLPVLDGLLHWAVCPSAEAQDPFSTLGPNAVLSPQRLVLETLSKLSIQDNNVDLILATPPFSRLEKLYSTMVRFLS
DRKNPVCREMAVVLLANLAQGDSLAARAIAVQKGSIGNLLGFLEDSLAATQFQQSQASLLHMQNPPFEPTSVDMMRR
AARALLALAKVDENHSEFTLYESRLLDISVSPLMNSLVSQVICDVLFLIGQS
```

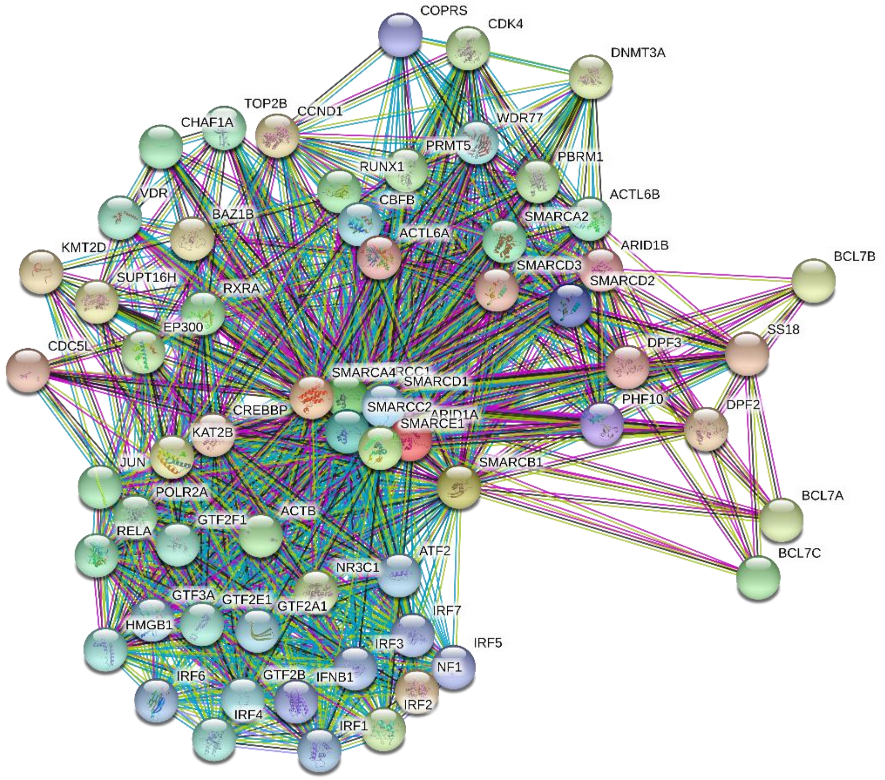

BAF250B/ARID1B (UniProt ID: Q8NFD5; PPID=76.9%; 2,236 residues)

```
>sp|Q8NFD5|ARI1B_HUMAN AT-rich interactive domain-containing protein 1B
OS=Homo sapiens OX=9606 GN=ARID1B PE=1 SV=2
MAHNAGAAAAAGTHSAKSGGSEAALKEGGSAAALSSSSSSSAAAAAASSSSSSGPGSAMETGLLPNHKLKTVGEAPA
APPHQQHHHHHAHHHHHHAHHLHHHHALQQQLNQFQQQQQQQQQQQQQQQQQQHPISNNNSLGGAGGGAPQPGPDM
EQPQHGGAKDSAAGGQADPPGPPLLSKPGDEDDAPPKMGEPAGGRYEHPGLGALGTQQPPVAVPGGGGGPAAVPEFN
NYYGSAAPASGGPGGRAGPCFDQHGGQQSPGMGMMHSASAAAAGAPGSMDPLQNSHEGYPNSQCNHYPGYSRPGAGG
GGGGGGGGGGSGGGGGGGGAGAGGAGAGAVAAAAAAAAAAAGGGGGGGYGGSSAGYGVLSSPRQQGGGMMMGPGGG
GAASLSKAAAGSAAGGFQRFAGQNQHPSGATPTLNQLLTSPSPMMRSYGGSYPEYSSPSAPPPPPSQPQSQAAAAGA
AAGGQQAAAGMGLGKDMGAQYAAASPAWAAAQQRSHPAMSPGTPGPTMGRSQGSPMDPMVMKRPQLYGMGSNPHSQP
QQSSPYPGGSYGPPGPQRYPIGIQGRTPGAMAGMQYPQQQMPPQYGQQGVSGYCQQGQQPYYSQQPQPPHLPPQAQY
LPSQSQQRYQPQQDMSQEGYGTRSQPPLAPGKPNHEDLNLIQQERPSSLPDLSGSIDDLPTGTEATLSSAVSASGST
SSQGDQSNPAQSPFSPHASPHLSSIPGGPSPSPVGSPVGSNQSRSGPISPASIPGSQMPPQPPGSQSESSSHPALSQ
SPMPQERGFMAGTQRNPQMAQYGPQQTGPSMSPHPSPGGQMHAGISSFQQSNSSGTYGPQMSQYGPQGNYSRPPAYS
GVPSASYSGPGPGMGISANNQMHGQGPSQPCGAVPLGRMPSAGMQNRPFPGNMSSMTPSSPGMSQQGGPGMGPPMPT
VNRKAQEAAAAVMQAAANSAQSRQGSFPGMNQSGLMASSSPYSQPMNNSSSLMNTQAPPYSMAPAMVNSSAASVGLA
DMMSPGESKLPLPLKADGKEEGTPQPESKSKKSSSSTTTGEKITKVYELGNEPERKLWVDRYLTFMEERGSPVSSLP
AVGKKPLDLFRLYVCVKEIGGLAQVNKNKKWRELATNLNVGTSSSAASSLKKQYIQYLFAFECKIERGEEPPPEVFS
TGDTKKQPKLQPPSPANSGSLQGPQTPQSTGSNSMAEVPGDLKPPTPASTPHGQMTPMQGGRSSTISVHDPFSDVSD
SSFPKRNSMTPNAPYQQGMSMPDVMGRMPYEPNKDPFGGMRKVPGSSEPFMTQGQMPNSSMQDMYNQSPSGAMSNLG
MGQRQQFPYGASYDRRHEPYGQQYPGQGPPSGQPPYGGHQPGLYPQQPNYKRHMDGMYGPPAKRHEGDMYNMQYSSQ
QQEMYNQYGGSYSGPDRRPIQGQYPYPYSRERMQGPGQIQTHGIPPQMMGGPLQSSSSEGPQQNMWAARNDMPYPYQ
NRQGPGGPTQAPPYPGMNRTDDMMVPDQRINHESQWPSHVSQRQPYMSSSASMQPITRPPQPSYQTPPSLPNHISRA
PSPASFQRSLENRMSPSKSPFLPSMKMQKVMPTVPTSQVTGPPPQPPPIRREITFPPGSVEASQPVLKQRRKITSKD
IVTPEAWRVMMSLKSGLLAESTWALDTINILLYDDSTVATFNLSQLSGFLELLVEYFRKCLIDIFGILMEYEVGDPS
QKALDHNAARKDDSQSLADDSGKEEEDAECIDDDEEDEEDEEEDSEKTESDEKSSIALTAPDAAADPKEKPKQASKF
DKLPIKIVKKNNLFVVDRSDKLGRVQEFNSGLLHWQLGGGDTTEHIQTHFESKMEIPPRRRPPPPLSSAGRKKEQEG
KGDSEEQQEKSIIATIDDVLSARPGALPEDANPGPQTESSKFPFGIQQAKSHRNIKLLEDEPRSRDETPLCTIAHWQ
DSLAKRCICVSNIVRSLSFVPGNDAEMSKHPGLVLILGKLILLHHEHPERKRAPQTYEKEEDEDKGVACSKDEWWWD
CLEVLRDNTLVTLANISGQLDLSAYTESICLPILDGLLHWMVCPSAEAQDPFPTVGPNSVLSPQRLVLETLCKLSIQ
DNNVDLILATPPFSRQEKFYATLVRYVGDRKNPVCREMSMALLSNLAQGDALAARAIAVQKGSIGNLISFLEDGVTM
AQYQQSQHNLMHMQPPPLEPPSVDMMCRAAKALLAMARVDENRSEFLLHEGRLLDISISAVLNSLVASVICDVLFQI
GQL
```

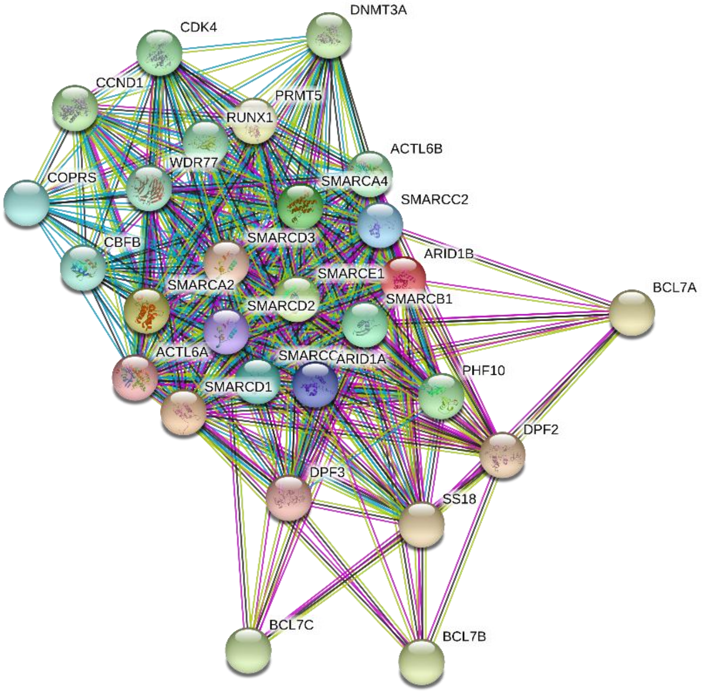

BAF57/SMARCE1 (UniProt ID: Q969G3; PPID=77.6%; 411 residues)

```
>sp|Q969G3|SMCE1_HUMAN SWI/SNF-related matrix-associated actin-dependent
regulator of chromatin subfamily E member 1 OS=Homo sapiens OX=9606
GN=SMARCE1 PE=1 SV=2
MSKRPSYAPPPTPAPATQMPSTPGFVGYNPYSHLAYNNYRLGGNPGTNSRVTASSGITIPKPPKPPDKPLMPYMRYS
RKVWDQVKASNPDLKLWEIGKIIGGMWRDLTDEEKQEYLNEYEAEKIEYNESMKAYHNSPAYLAYINAKSRAEAALE
EESRQRQSRMEKGEPYMSIQPAEDPDDYDDGFSMKHTATARFQRNHRLISEILSESVVPDVRSVVTTARMQVLKRQV
QSLMVHQRKLEAELLQIEERHQEKKRKFLESTDSFNNELKRLCGLKVEVDMEKIAAEIAQAEEQARKRQEEREKEAA
EQAERSQSSIVPEEEQAANKGEEKKDDENIPMETEETHLEETTESQQNGEEGTSTPEDKESGQEGVDSMAEEGTSDS
NTGSESNSATVEEPPTDPIPEDEKKE
```

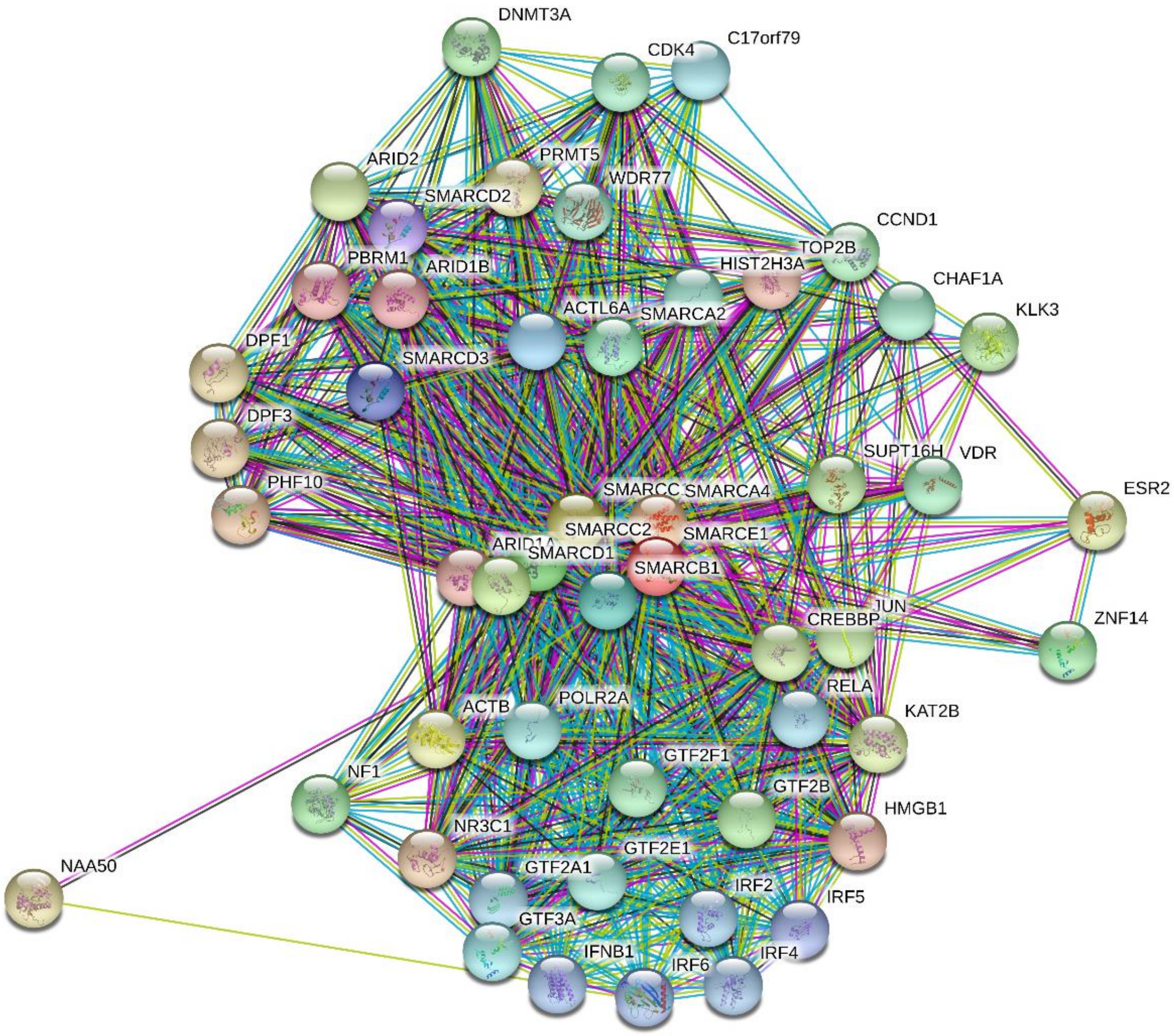

BAF155/SMARCC1 (UniProt ID: Q92922; PPID=59.2%; 1,105 residues)

```
>sp|Q92922|SMRC1_HUMAN SWI/SNF complex subunit SMARCC1 OS=Homo sapiens
OX=9606 GN=SMARCC1 PE=1 SV=3
MAAAAGGGGPGTAVGATGSGIAAAAAGLAVYRRKDGGPATKFWESPETVSQLDSVRVWLGKHYKKYVHADAPTNKTL
AGLVVQLLQFQEDAFGKHVTNPAFTKLPAKCFMDFKAGGALCHILGAAYKYKNEQGWRRFDLQNPSRMDRNVEMFMN
IEKTLVQNNCLTRPNIYLIPDIDLKLANKLKDIIKRHQGTFTDEKSKASHHIYPYSSSQDDEEWLRPVMRKEKQVLV
HWGFYPDSYDTWVHSNDVDAEIEDPPIPEKPWKVHVKWILDTDIFNEWMNEEDYEVDENRKPVSFRQRISTKNEEPV
RSPERRDRKASANARKRKHSPSPPPPTPTESRKKSGKKGQASLYGKRRSQKEEDEQEDLTKDMEDPTPVPNIEEVVL
PKNVNLKKDSENTPVKGGTVADLDEQDEETVTAGGKEDEDPAKGDQSRSVDLGEDNVTEQTNHIIIPSYASWFDYNC
IHVIERRALPEFFNGKNKSKTPEIYLAYRNFMIDTYRLNPQEYLTSTACRRNLTGDVCAVMRVHAFLEQWGLVNYQV
DPESRPMAMGPPPTPHFNVLADTPSGLVPLHLRSPQVPAAQQMLNFPEKNKEKPVDLQNFGLRTDIYSKKTLAKSKG
ASAGREWTEQETLLLLEALEMYKDDWNKVSEHVGSRTQDECILHFLRLPIEDPYLENSDASLGPLAYQPVPFSQSGN
PVMSTVAFLASVVDPRVASAAAKAALEEFSRVREEVPLELVEAHVKKVQEAARASGKVDPTYGLESSCIAGTGPDEP
EKLEGAEEEKMEADPDGQQPEKAENKVENETDEGDKAQDGENEKNSEKEQDSEVSEDTKSEEKETEENKELTDTCKE
RESDTGKKKVEHEISEGNVATAAAAALASAATKAKHLAAVEERKIKSLVALLVETQMKKLEIKLRHFEELETIMDRE
KEALEQQRQQLLTERQNFHMEQLKYAELRARQQMEQQQHGQNPQQAHQHSGGPGLAPLGAAGHPGMMPHQQPPPYPL
MHHQMPPPHPPQPGQIPGPGSMMPGQHMPGRMIPTVAANIHPSGSGPTPPGMPPMPGNILGPRVPLTAPNGMYPPPP
QQQPPPPPPPADGVPPPPAPGPPASAAP
```

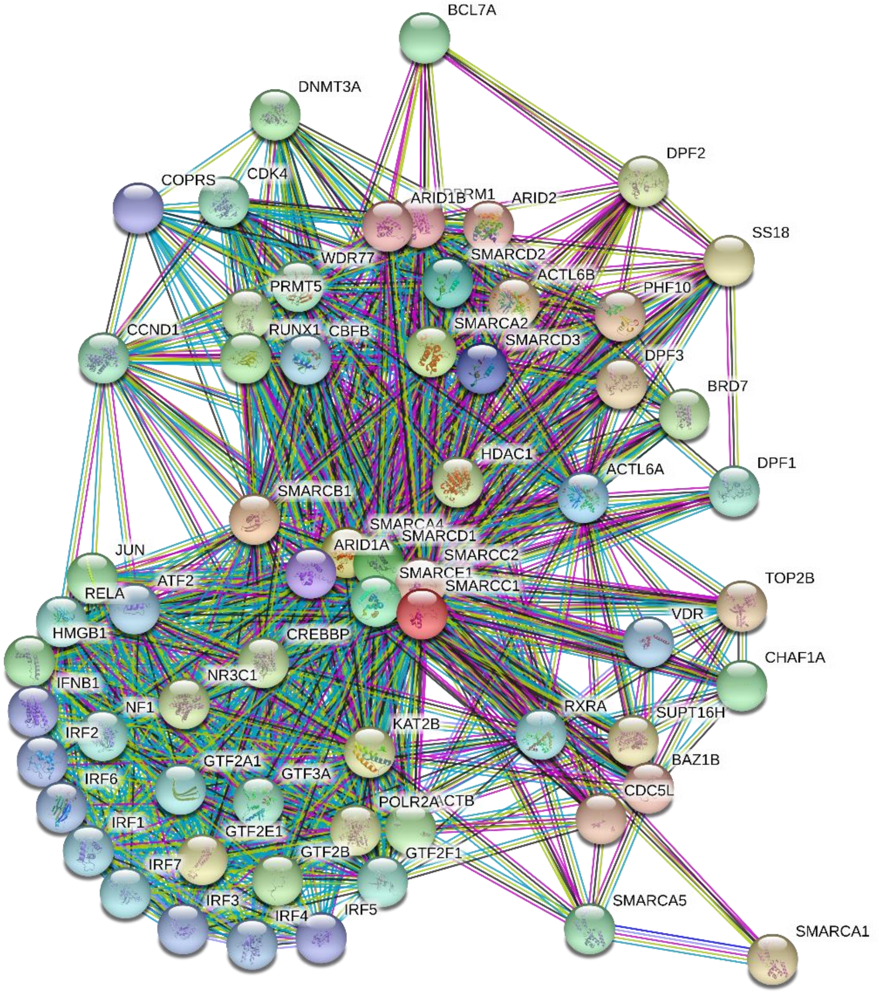

BAF180/PBRM1 (UniProt ID: Q86U86; PPID=43.2%; 1,689 residues)

```
>sp|Q86U86|PB1_HUMAN Protein polybromo-1 OS=Homo sapiens OX=9606 GN=PBRM1
PE=1 SV=1
MGSKRRRATSPSSSVSGDFDDGHHSVSTPGPSRKRRRLSNLPTVDPIAVCHELYNTIRDYKDEQGRLLCELFIRAPK
RRNQPDYYEVVSQPIDLMKIQQKLKMEEYDDVNLLTADFQLLFNNAKSYYKPDSPEYKAACKLWDLYLRTRNEFVQK
GEADDEDDDEDGQDNQGTVTEGSSPAYLKEILEQLLEAIVVATNPSGRLISELFQKLPSKVQYPDYYAIIKEPIDLK
TIAQRIQNGSYKSIHAMAKDIDLLAKNAKTYNEPGSQVFKDANSIKKIFYMKKAEIEHHEMAKSSLRMRTPSNLAAA
RLTGPSHSKGSLGEERNPTSKYYRNKRAVQGGRLSAITMALQYGSESEEDAALAAARYEEGESEAESITSFMDVSNP
FYQLYDTVRSCRNNQGQLIAEPFYHLPSKKKYPDYYQQIKMPISLQQIRTKLKNQEYETLDHLECDLNLMFENAKRY
NVPNSAIYKRVLKLQQVMQAKKKELARRDDIEDGDSMISSATSDTGSAKRKSKKNIRKQRMKILFNVVLEAREPGSG
RRLCDLFMVKPSKKDYPDYYKIILEPMDLKIIEHNIRNDKYAGEEGMIEDMKLMFRNARHYNEEGSQVYNDAHILEK
LLKEKRKELGPLPDDDDMASPKLKLSRKSGISPKKSKYMTPMQQKLNEVYEAVKNYTDKRGRRLSAIFLRLPSRSEL
PDYYLTIKKPMDMEKIRSHMMANKYQDIDSMVEDFVMMFNNACTYNEPESLIYKDALVLHKVLLETRRDLEGDEDSH
VPNVTLLIQELIHNLFVSVMSHQDDEGRCYSDSLAEIPAVDPNFPNKPPLTFDIIRKNVENNRYRRLDLFQEHMFEV
LERARRMNRTDSEIYEDAVELQQFFIKIRDELCKNGEILLSPALSYTTKHLHNDVEKERKEKLPKEIEEDKLKREEE
KREAEKSEDSSGAAGLSGLHRTYSQDCSFKNSMYHVGDYVYVEPAEANLQPHIVCIERLWEDSAGEKWLYGCWFYRP
NETFHLATRKFLEKEVFKSDYYNKVPVSKILGKCVVMFVKEYFKLCPENFRDEDVFVCESRYSAKTKSFKKIKLWTM
PISSVRFVPRDVPLPVVRVASVFANADKGDDEKNTDNSEDSRAEDNFNLEKEKEDVPVEMSNGEPGCHYFEQLHYND
MWLKVGDCVFIKSHGLVRPRVGRIEKVWVRDGAAYFYGPIFIHPEETEHEPTKMFYKKEVFLSNLEETCPMTCILGK
CAVLSFKDFLSCRPTEIPENDILLCESRYNESDKQMKKFKGLKRFSLSAKVVDDEIYYFRKPIVPQKEPSPLLEKKI
QLLEAKFAELEGGDDDIEEMGEEDSEVIEPPSLPQLQTPLASELDLMPYTPPQSTPKSAKGSAKKEGSKRKINMSGY
ILFSSEMRAVIKAQHPDYSFGELSRLVGTEWRNLETAKKAEYEERAAKVAEQQERERAAQQQQPSASPRAGTPVGAL
MGVVPPPTPMGMLNQQLTPVAGMMGGYPPGLPPLQGPVDGLVSMGSMQPLHPGGPPPHHLPPGVPGLPGIPPPGVMN
QGVAPMVGTPAPGGSPYGQQVGVLGPPGQQAPPPYPGPHPAGPPVIQQPTTPMFVAPPPKTQRLLHSEAYLKYIEGL
SAESNSISKWDQTLAARRRDVHLSKEQESRLPSHWLKSKGAHTTMADALWRLRDLMLRDTLNIRQAYNLENV
```

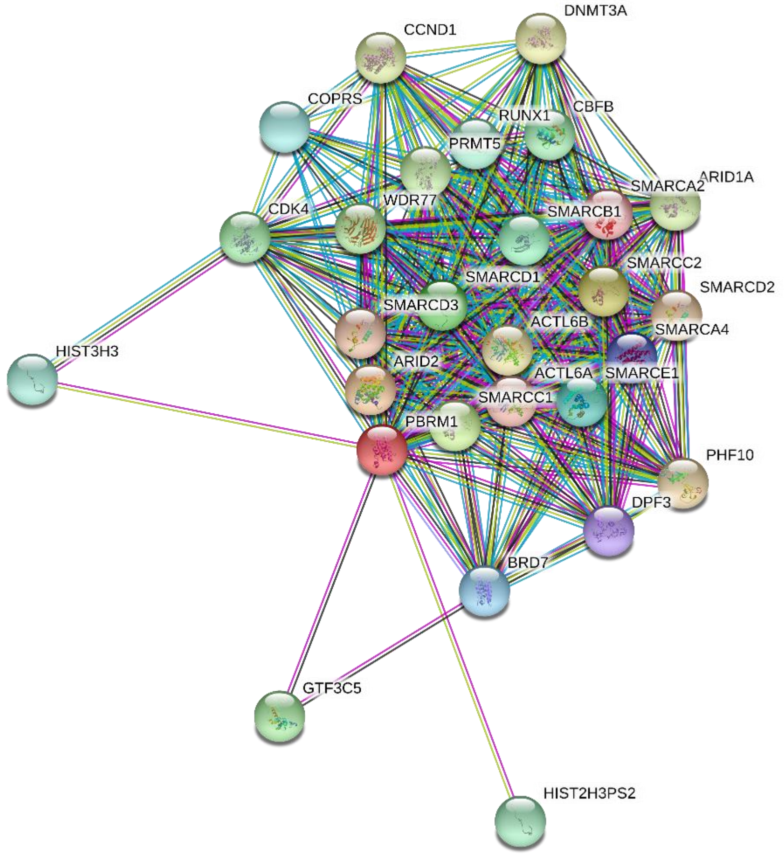

BCL7A (UniProt ID: Q4VC05; PPID= 87.6%; 210 residues)

```
>sp|Q4VC05|BCL7A_HUMAN B-cell CLL/lymphoma 7 protein family member A OS=Homo
sapiens OX=9606 GN=BCL7A PE=1 SV=1
MSGRSVRAETRSRAKDDIKRVMAAIEKVRKWEKKWVTVGDTSLRIYKWVPVTEPKVDDKNKNKKKGKDEKCGSEVTT
PENSSSPGMMDMHDDNSNQSSIADASPIKQENSSNSSPAPEPNSAVPSDGTEAKVDEAQADGKEHPGAEDASDEQNS
QSSMEHSMNSSEKVDRQPSGDSGLAAETSAISQDLEGVPPSKKMKLEASQQNSEEM
```

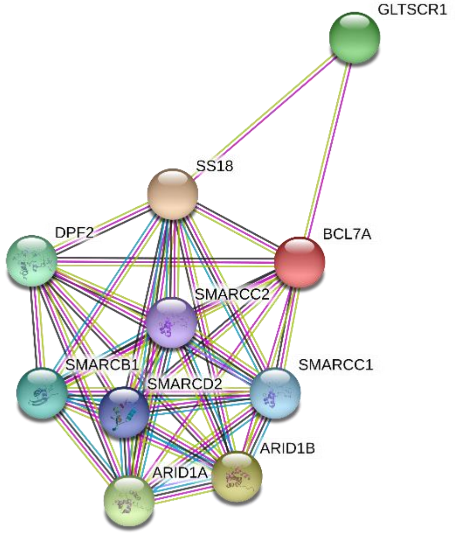

BCL7B (UniProt ID: Q9BQE9; PPID=89.6%; 202 residues)

```
>sp|Q9BQE9|BCL7B_HUMAN B-cell CLL/lymphoma 7 protein family member B OS=Homo
sapiens OX=9606 GN=BCL7B PE=1 SV=1
MSGRSVRAETRSRAKDDIKKVMAAIEKVRKWEKKWVTVGDTSLRIFKWVPVTDSKEKEKSKSNSSAAREPNGFPSDA
SANSSLLLEFQDENSNQSSVSDVYQLKVDSSTNSSPSPQQSESLSPAHTSDFRTDDSQPPTLGQEILEEPSLPSSEV
ADEPPTLTKEEPVPLETQVVEEEEDSGAPPLKRFCVDQPTVPQTASES
```

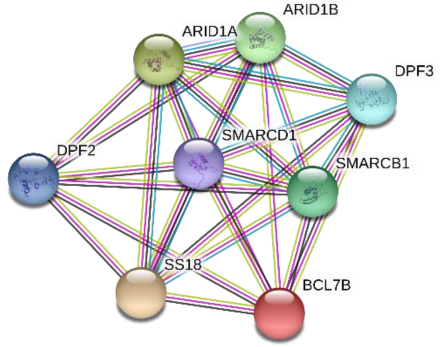

BCL7C (UniProt ID: Q8WUZ0; PPID=90.0%; 217 residues)

```
>sp|Q8WUZ0|BCL7C_HUMAN B-cell CLL/lymphoma 7 protein family member C OS=Homo
sapiens OX=9606 GN=BCL7C PE=1 SV=3
MAGRTVRAETRSRAKDDIKKVMATIEKVRRWEKRWVTVGDTSLRIFKWVPVVDPQEEERRRAGGGAERSRGRERRGR
GASPRGGGPLILLDLNDENSNQSFHSEGSLQKGTEPSPGGTPQPSRPVSPAGPPEGVPEEAQPPRLGQERDPGGITA
GSTDEPPMLTKEEPVPELLEAEAPEAYPVFEPVPPVPEAAQGDTEDSEGAPPLKRICPNAPDP
```

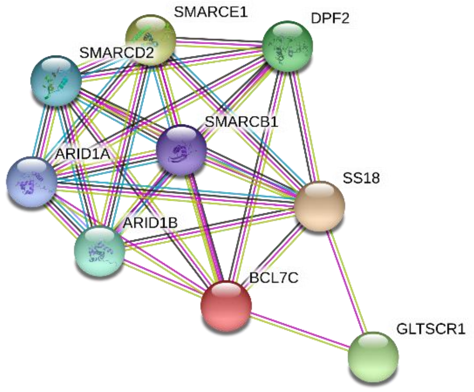

BCL11A (UniProt ID: Q9H165; PPID=69.0%; 835 residues)

```
>sp|Q9H165|BC11A_HUMAN B-cell lymphoma/leukemia 11A OS=Homo sapiens OX=9606
GN=BCL11A PE=1 SV=2
MSRRKQGKPQHLSKREFSPEPLEAILTDDEPDHGPLGAPEGDHDLLTCGQCQMNFPLGDILIFIEHKRKQCNGSLCL
EKAVDKPPSPSPIEMKKASNPVEVGIQVTPEDDDCLSTSSRGICPKQEHIADKLLHWRGLSSPRSAHGALIPTPGMS
AEYAPQGICKDEPSSYTCTTCKQPFTSAWFLLQHAQNTHGLRIYLESEHGSPLTPRVGIPSGLGAECPSQPPLHGIH
IADNNPFNLLRIPGSVSREASGLAEGRFPPTPPLFSPPPRHHLDPHRIERLGAEEMALATHHPSAFDRVLRLNPMAM
EPPAMDFSRRLRELAGNTSSPPLSPGRPSPMQRLLQPFQPGSKPPFLATPPLPPLQSAPPPSQPPVKSKSCEFCGKT
FKFQSNLVVHRRSHTGEKPYKCNLCDHACTQASKLKRHMKTHMHKSSPMTVKSDDGLSTASSPEPGTSDLVGSASSA
LKSVVAKFKSENDPNLIPENGDEEEEEDDEEEEEEEEEEEEEELTESERVDYGFGLSLEAARHHENSSRGAVVGVGDE
SRALPDVMQGMVLSSMQHFSEAFHQVLGEKHKRGHLAEAEGHRDTCDEDSVAGESDRIDDGTVNGRGCSPGESASGG
LSKKLLLGSPSSLSPFSKRIKLEKEFDLPPAAMPNTENVYSQWLAGYAASRQLKDPFLSFGDSRQSPFASSSEHSSE
NGSLRFSTPPGELDGGISGRSGTGSGGSTPHISGPGPGRPSSKEGRRSDTCEYCGKVFKNCSNLTVHRRSHTGERPY
KCELCNYACAQSSKLTRHMKTHGQVGKDVYKCEICKMPFSVYSTLEKHMKKWHSDRVLNNDIKTE
```

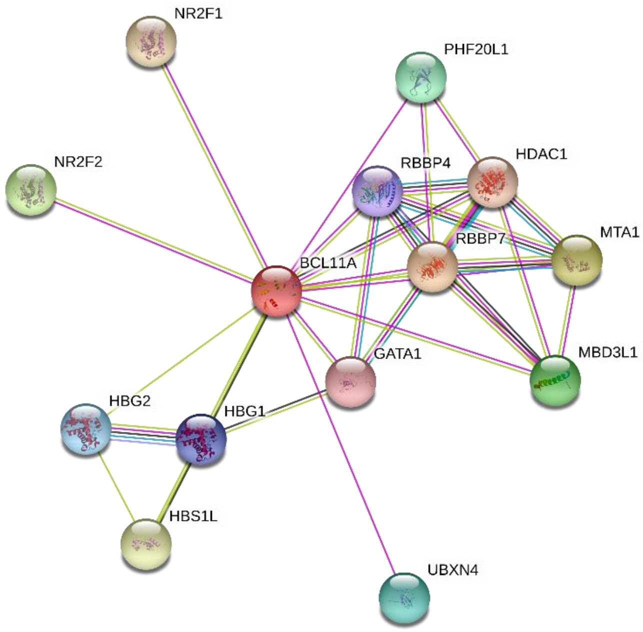

BCL11B (UniProt ID: Q9C0K0; PPID=69.8%; 894 residues)

>sp|Q9C0K0|BC11B_HUMAN B-cell lymphoma/leukemia 11B OS=Homo sapiens OX=9606
GN=BCL11B PE=1 SV=1
MSRRKQGNPQHLSQRELITPEADHVEAAILEEDEGLEIEEPSGLGLMVGGPDPDLLTCGQCQMNFPLGDILVFIEHK
RKQCGGSLGACYDKALDKDSPPPSSRSELRKVSEPVEIGIQVTPDEDDHLLSPTKGICPKQENIAGPCRPAQLPAVA
PIAASSHPHSSVITSPLRALGALPPCLPLPCCSARPVSGDGTQGEGQTEAPFGCQCQLSGKDEPSSYICTTCKQPFN
SAWFLLQHAQNTHGFRIYLEPGPASSSLTPRLTIPPPLGPEAVAQSPLMNFLGDSNPFNLLRMTGPILRDHPGFGEG
RLPGTPPLFSPPPRHHLDPHRLSAEEMGLVAQHPSAFDRVMRLNPMAIDSPAMDFSRRLRELAGNSSTPPPVSPGRG
NPMHRLLNPFQPSPKSPFLSTPPLPPMPPGGTPPPQPPAKSKSCEFCGKTFKFQSNLIVHRRSHTGEKPYKCQLCDH
ACSQASKLKRHMKTHMHKAGSLAGRSDDGLSAASSPEPGTSELAGEGLKAADGDFRHHESDPSLGHEPEEEDEEEEE
EEEELLLENESRPESSFSMDSELSRNRENGGGGVPGVPGAGGGAAKALADEKALVLGKVMENVGLGALPQYGELLAD
KQKRGAFLKRAAGGGDAGDDDDAGGCGDAGAGGAVNGRGGGFAPGTEPFPGLFPRKPAPLPSPGLNSAAKRIKVEKD
LELPPAALIPSENVYSQWLVGYAASRHFMKDPFLGFTDARQSPFATSSEHSSENGSLRFSTPPGDLLDGGLSGRSGT
ASGGSTPHLGGPGPGRPSSKEGRRSDTCEYCGKVFKNCSNLTVHRRSHTGERPYKCELCNYACAQSSKLTRHMKTHG
QIGKEVYRCDICQMPFSVYSTLEKHMKKWHGEHLLTNDVKIEQAERS

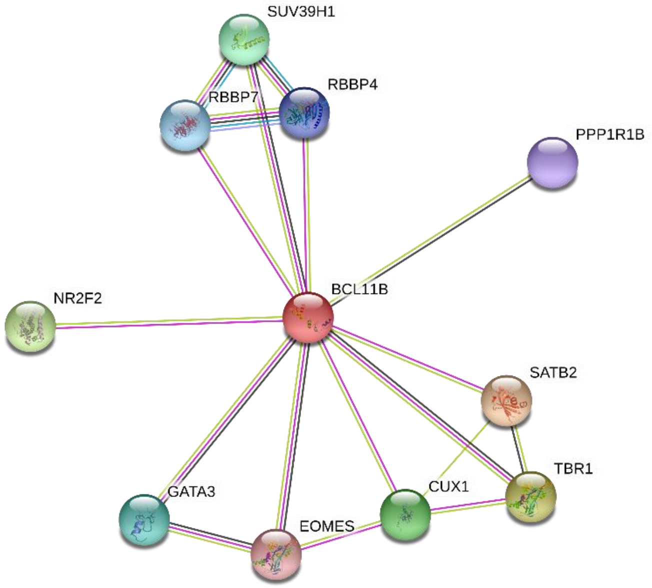

SSXT/SS18 (UniProt ID: Q15532; PPID= 84.1%; 418 residues)

```
>sp|Q15532|SSXT_HUMAN Protein SSXT OS=Homo sapiens OX=9606 GN=SS18 PE=1 SV=3
MSVAFAAPRQRGKGEITPAAIQKMLDDNNHLIQCIMDSQNKGKTSECSQYQQMLHTNLVYLATIADSNQNMQSLLPA
PPTQNMPMGPGGMNQSGPPPPPRSHNMPSDGMVGGGPPAPHMQNQMNGQMPGPNHMPMQGPGPNQLNMTNSSMNMPS
SSHGSMGGYNHSVPSSQSMPVQNQMTMSQGQPMGNYGPRPNMSMQPNQGPMMHQQPPSQQYNMPQGGGQHYQGQQPP
MGMMGQVNQGNHMMGQRQIPPYRPPQQGPPQQYSGQEDYYGDQYSHGGQGPPEGMNQQYYPDGHNDYGYQQPSYPEQ
GYDRPYEDSSQHYYEGGNSQYGQQQDAYQGPPPQQGYPPQQQQYPGQQGYPGQQQGYGPSQGGPGPQYPNYPQGQGQ
QYGGYRPTQPGPPQPPQQRPYGYDQGQYGNYQQ
```

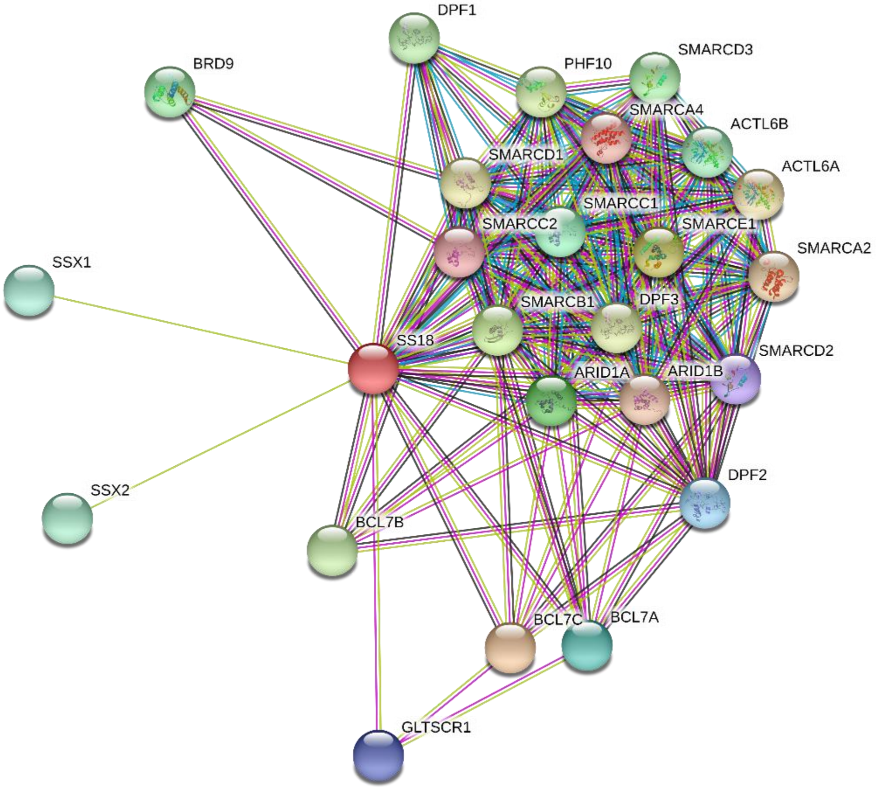

BAF200/ARID2 (UniProt ID: Q68CP9; PPID= 61.3%; 1,835 residues)

```
>sp|Q68CP9|ARID2_HUMAN AT-rich interactive domain-containing protein 2
OS=Homo sapiens OX=9606 GN=ARID2 PE=1 SV=2
MANSTGKAPPDERRKGLAFLDELRQFHHSRGSPFKKIPAVGGKELDLHGLYTRVTTLGGFAKVSEKNQWGEIVEEFN
FPRSCSNAAFALKQYYLRYLEKYEKVHHFGEDDDEVPPGNPKPQLPIGAIPSSYNYQQHSVSDYLRQSYGLSMDFNS
PNDYNKLVLSLLSGLPNEVDFAINVCTLLSNESKHVMQLEKDPKIITLLLANAGVFDDTLGSFSTVFGEEWKEKTDR
DFVKFWKDIVDDNEVRDLISDRNKSHEGTSGEWIWESLFHPPRKLGINDIEGQRVLQIAVILRNLSFEEGNVKLLAA
NRTCLRFLLLSAHSHFISLRQLGLDTLGNIAAELLLDPVDFKTTHLMFHTVTKCLMSRDRFLKMRGMEILGNLCKAE
DNGVLICEYVDQDSYREIICHLTLPDVLLVISTLEVLYMLTEMGDVACTKIAKVEKSIDMLVCLVSMDIQMFGPDAL
AAVKLIEHPSSSHQMLSEIRPQAIEQVQTQTHVASAPASRAVVAQHVAPPPGIVEIDSEKFACQWLNAHFEVNPDCS
VSRAEMYSEYLSTCSKLARGGILTSTGFYKCLRTVFPNHTVKRVEDSSSNGQAHIHVVGVKRRAIPLPIQMYYQQQP
VSTSVVRVDSVPDVSPAPSPAGIPHGSQTIGNHFQRTPVANQSSNLTATQMSFPVQGVHTVAQTVSRIPQNPSPHTH
QQQNAPVTVIQSKAPIPCEVVKATVIQNSIPQTGVPVSIAVGGGPPQSSVVQNHSTGPQPVTVVNSQTLLHHPSVIP
QQSPLHTVVPGQIPSGTPVTVIQQAVPQSHMFGRVQNIPACTSTVSQGQQLITTSPQPVQTSSQQTSAGSQSQDTVI
IAPPQYVTTSASNIVSATSVQNFQVATGQMVTIAGVPSPQASRVGFQNIAPKPLPSQQVSSTVVQQPIQQPQQPTQQ
SVVIVSQPAQQGQTYAPAIHQIVLANPAALPAGQTVQLTGQPNITPSSSPSPVPATNNQVPTAMSSSSTPQSQGPPP
TVSQMLSVKRQQQQQHSPAPPPQQVQVQVQQPQQVQMQVQPQQSNAGVGQPASGESSLIKQLLLPKRGPSTPGGKLI
LPAPQIPPPNNARAPSPQVVYQVASNQAAGFGVQGQTPAQQLLVGQQNVQLVPSAMPPSGGVQTVPISNLQILPGPL
ISNSPATIFQGTSGNQVTITVVPNTSFAPATVSQGNATQLIAPAGITMSGTQTGVGLPVQTLPATQASPAGQSSCTT
ATPPFKGDKIICQKEEEAKEATGLHVHERKIEVMENPSCRRGATNTSNGDTKENEMHVGSLLNGRKYSDSSLPPSNS
GKIQSETNQCSLISNGPSLELGENGASGKQNSEQIDMQDIKSDLRKPLVNGICDFDKGDGSHLSKNIPNHKTSNHVG
NGEISPMEPQGTLDITQQDTAKGDQLERISNGPVLTLGGSSVSSIQEASNAATQQFSGTDLLNGPLASSLNSDVPQQ
RPSVVVSPHSTTSVIQGHQIIAVPDSGSKVSHSPALSSDVRSTNGTAECKTVKRPAEDTDRETVAGIPNKVGVRIVT
ISDPNNAGCSATMVAVPAGADPSTVAKVAIESAVQQKQQHPPTYVQNVVPQNTPMPPSPAVQVQGQPNSSQPSPFSG
SSQPGDPMRKPGQNFMCLWQSCKKWFQTPSQVFYHAATEHGGKDVYPGQCLWEGCEPFQRQRFSFITHLQDKHCSKD
ALLAGLKQDEPGQAGSQKSSTKQPTVGGTSSTPRAQKAIVNHPSAALMALRRGSRNLVFRDFTDEKEGPITKHIRLT
AALILKNIGKYSECGRRLLKRHENNLSVLAISNMEASSTLAKCLYELNFTVQSKEQEKDSEMLQ
```

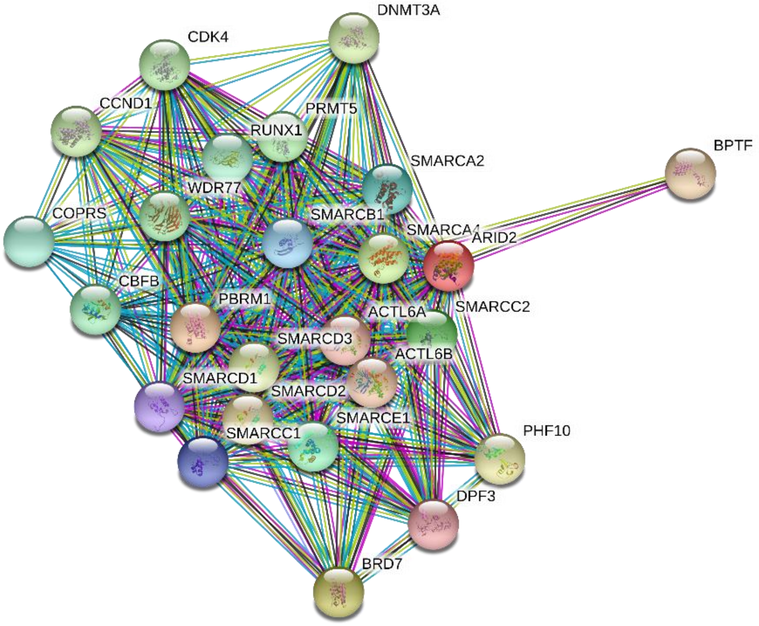

BRD7 (UniProt ID: Q9NPI1; PPID= 61.2%; 651 residues)

```
>sp|Q9NPI1|BRD7_HUMAN Bromodomain-containing protein 7 OS=Homo sapiens
OX=9606 GN=BRD7 PE=1 SV=1
MGKKHKKHKSDKHLYEEYVEKPLKLVLKVGGNEVTELSTGSSGHDSSLFEDKNDHDKHKDRKRKKRKKGEKQIPGEE
KGRKRRRVKEDKKKRDRDRVENEAEKDLQCHAPVRLDLPPEKPLTSSLAKQEEVEQTPLQEALNQLMRQLQRKDPSA
FFSFPVTDFIAPGYSMIIKHPMDFSTMKEKIKNNDYQSIEELKDNFKLMCTNAMIYNKPETIYYKAAKKLLHSGMKI
LSQERIQSLKQSIDFMADLQKTRKQKDGTDTSQSGEDGGCWQREREDSGDAEAHAFKSPSKENKKKDKDMLEDKFKS
NNLEREQEQLDRIVKESGGKLTRRLVNSQCEFERRKPDGTTTLGLLHPVDPIVGEPGYCPVRLGMTTGRLQSGVNTL
QGFKEDKRNKVTPVLYLNYGPYSSYAPHYDSTFANISKDDSDLIYSTYGEDSDLPSDFSIHEFLATCQDYPYVMADS
LLDVLTKGGHSRTLQEMEMSLPEDEGHTRTLDTAKEMEITEVEPPGRLDSSTQDRLIALKAVTNFGVPVEVFDSEEA
EIFQKKLDETTRLLRELQEAQNERLSTRPPPNMICLLGPSYREMHLAEQVTNNLKELAQQVTPGDIVSTYGVRKAMG
ISIPSPVMENNFVDLTEDTEEPKKTDVAECGPGGS
```

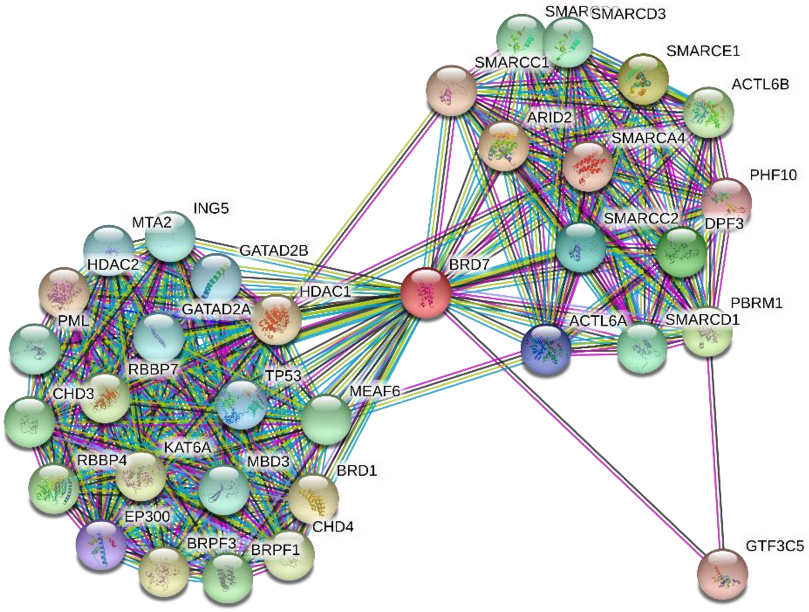

BRD9 (UniProt ID: Q9H8M2; PPID= 60.1%; 597 residues)

```
>sp|Q9H8M2|BRD9_HUMAN Bromodomain-containing protein 9 OS=Homo sapiens
OX=9606 GN=BRD9 PE=1 SV=2
MGKKHKKHKAEWRSSYEDYADKPLEKPLKLVLKVGGSEVTELSGSGHDSSYYDDRSDHERERHKEKKKKKKKKSEKE
KHLDDEERRKRKEEKKRKREREHCDTEGEADDFDPGKKVEVEPPPDRPVRACRTQPAENESTPIQQLLEHFLRQLQR
KDPHGFFAFPVTDAIAPGYSMIIKHPMDFGTMKDKIVANEYKSVTEFKADFKLMCDNAMTYNRPDTVYYKLAKKILH
AGFKMMSKQAALLGNEDTAVEEPVPEVVPVQVETAKKSKKPSREVISCMFEPEGNACSLTDSTAEEHVLALVEHAAD
EARDRINRFLPGGKMGYLKRNGDGSLLYSVVNTAEPDADEEETHPVDLSSLSSKLLPGFTTLGFKDERRNKVTFLSS
ATTALSMQNNSVFGDLKSDEMELLYSAYGDETGVQCALSLQEFVKDAGSYSKKVVDDLLDQITGGDHSRTLFQLKQR
RNVPMKPPDEAKVGDTLGDSSSSVLEFMSMKSYPDVSVDISMLSSLGKVKKELDPDDSHLNLDETTKLLQDLHEAQA
ERGGSRPSSNLSSLSNASERDQHHLGSPSRLSVGEQPDVTHDPYEFLQSPEPAASAKT
```

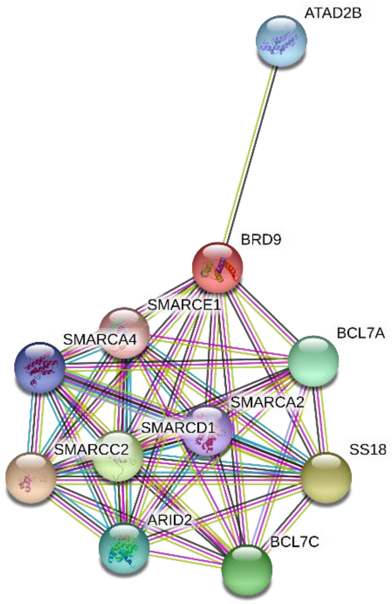

BAF60A/SMARCD1 (UniProt ID: Q96GM5; PPID= 43.8%; 515 residues)

>sp|Q96GM5|SMRD1_HUMAN SWI/SNF-related matrix-associated actin-dependent regulator of chromatin subfamily D member 1 OS=Homo sapiens OX=9606 GN=SMARCD1 PE=1 SV=2
MAARAGFQSVAPSGGAGASGGAGAAAALGPGGTPGPPVRMGPAPGQGLYRSPMPGAAYPRPGMLPGSRMTPQGPSMG
PPGYGGNPSVRPGLAQSGMDQSRKRPAPQQIQQVQQQAVQNRNHNAKKKKMADKILPQRIRELVPESQAYMDLLAFE
RKLDQTIMRKRLDIQEALKRPIKQKRKLRIFISNTFNPAKSDAEDGEGTVASWELRVEGRLLEDSALSKYDATKQKR
KFSSFFKSLVIELDKDLYGPDNHLVEWHRTATTQETDGFQVKRPGDVNVRCTVLLMLDYQPPQFKLDPRLARLLGIH
TQTRPVIIQALWQYIKTHKLQDPHEREFVICDKYLQQIFESQRMKFSEIPQRLHALLMPPEPIIINHVISVDPNDQK
KTACYDIDVEVDDTLKTQMNSFLLSTASQQEIATLDNKIHETIETINQLKTQREFMLSFARDPQGFINDWLQSQCRD
LKTMTDVVGNPEEERRAEFYFQPWAQEAVCRYFYSKVQQRRQELEQALGIRNT

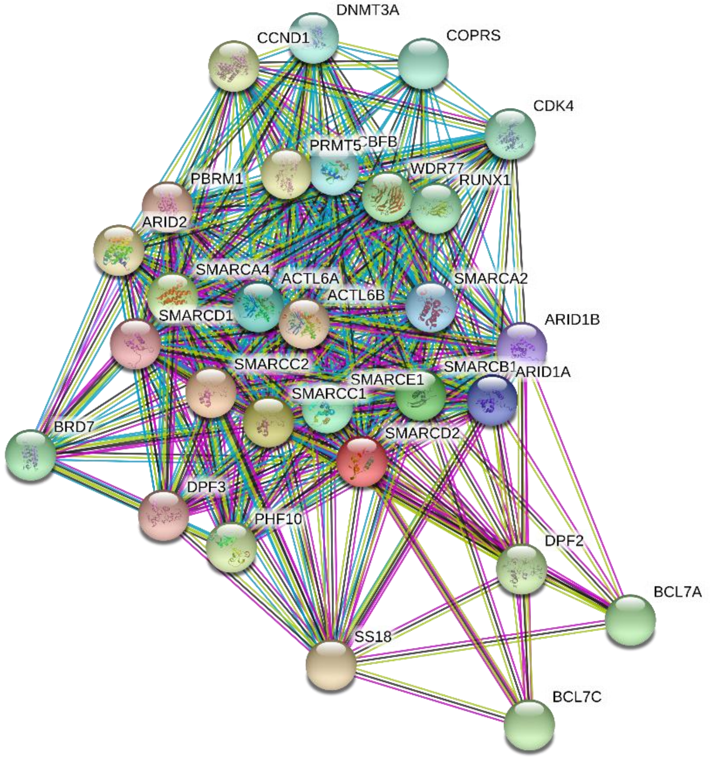

BAF60B/SMARCD2 (UniProt ID: Q92925; PPID= 49.3%; 531 residues)

```
>sp|Q92925|SMRD2_HUMAN SWI/SNF-related matrix-associated actin-dependent
regulator of chromatin subfamily D member 2 OS=Homo sapiens OX=9606
GN=SMARCD2 PE=1 SV=3
MSGRGAGGFPLPPLSPGGGAVAAALGAPPPPAGPGMLPGPALRGPGPAGGVGGPGAAAFRPMGPAGPAAQYQRPGMS
PGNRMPMAGLQVGPPAGSPFGAAAPLRPGMPPTMMDPFRKRLLVPQAQPPMPAQRRGLKRRKMADKVLPQRIRELVP
ESQAYMDLLAFERKLDQTIARKRMEIQEAIKKPLTQKRKLRIYISNTFSPSKAEGDSAGTAGTPGGTPAGDKVASWE
LRVEGKLLDDPSKQKRKFSSFFKSLVIELDKELYGPDNHLVEWHRMPTTQETDGFQVKRPGDLNVKCTLLLMLDHQP
PQYKLDPRLARLLGVHTQTRAAIMQALWLYIKHNQLQDGHEREYINCNRYFRQIFSCGRLRFSEIPMKLAGLLQHPD
PIVINHVISVDPNDQKKTACYDIDVEVDDPLKAQMSNFLASTTNQQEIASLDVKIHETIESINQLKTQRDFMLSFST
DPQDFIQEWLRSQRRDLKIITDVIGNPEEERRAAFYHQPWAQEAVGRHIFAKVQQRRQELEQVLGIRLT
```

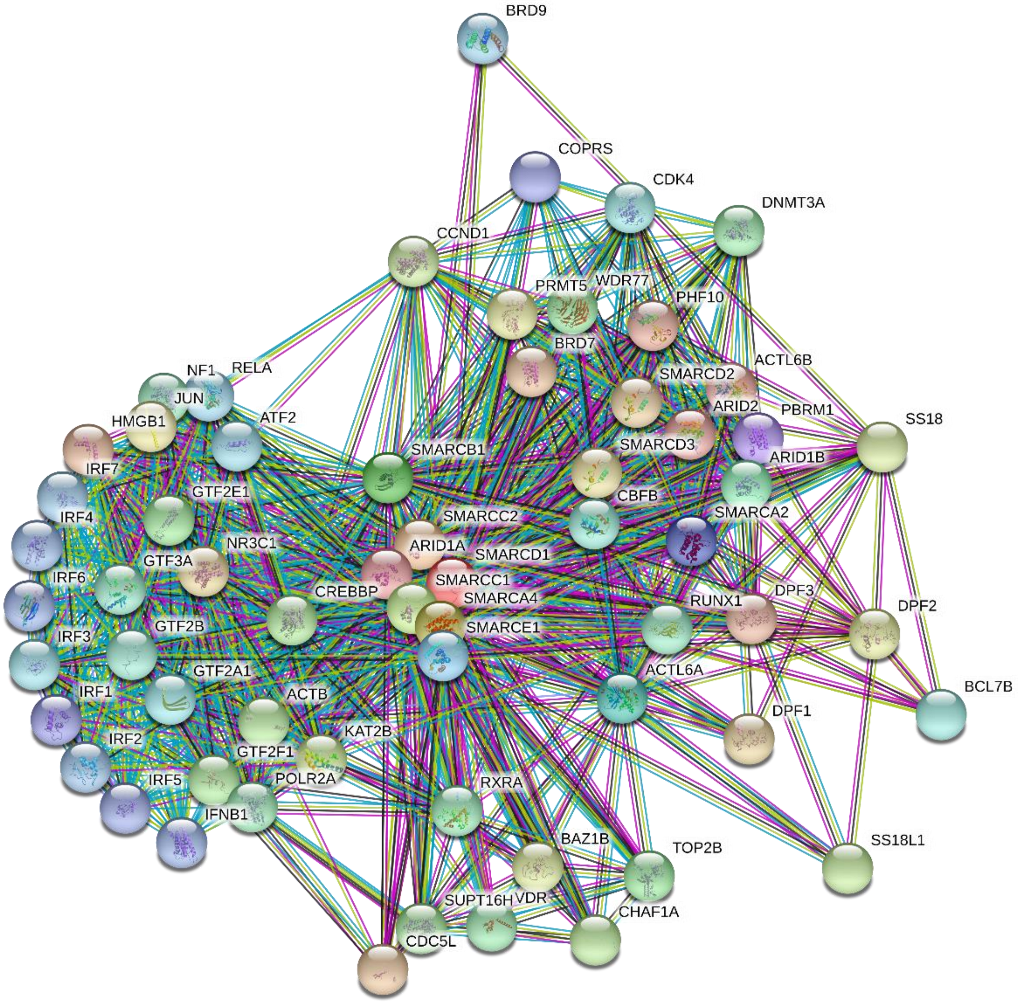

BAF60C/SMARCD3 (UniProt ID: Q6STE5; PPID=50.5%; 483 residues)

```
>sp|Q6STE5|SMRD3_HUMAN SWI/SNF-related matrix-associated actin-dependent
regulator of chromatin subfamily D member 3 OS=Homo sapiens OX=9606
GN=SMARCD3 PE=1 SV=1
MAADEVAGGARKATKSKLFEFLVHGVRPGMPSGARMPHQGAPMGPPGSPYMGSPAVRPGLAPAGMEPARKRAAPPPG
QSQAQSQGQPVPTAPARSRSAKRRKMADKILPQRIRELVPESQAYMDLLAFERKLDQTIMRKRVDIQEALKRPMKQK
RKLRLYISNTFNPAKPDAEDSDGSIASWELRVEGKLLDDPSKQKRKFSSFFKSLVIELDKDLYGPDNHLVEWHRTPT
TQETDGFQVKRPGDLSVRCTLLLMLDYQPPQFKLDPRLARLLGLHTQSRSAIVQALWQYVKTNRLQDSHDKEYINGD
KYFQQIFDCPRLKFSEIPQRLTALLLPPDPIVINHVISVDPSDQKKTACYDIDVEVEEPLKGQMSSFLLSTANQQEI
SALDSKIHETIESINQLKIQRDFMLSFSRDPKGYVQDLLRSQSRDLKVMTDVAGNPEEERRAEFYHQPWSQEAVSRY
FYCKIQQRRQELEQSLVVRNT
```

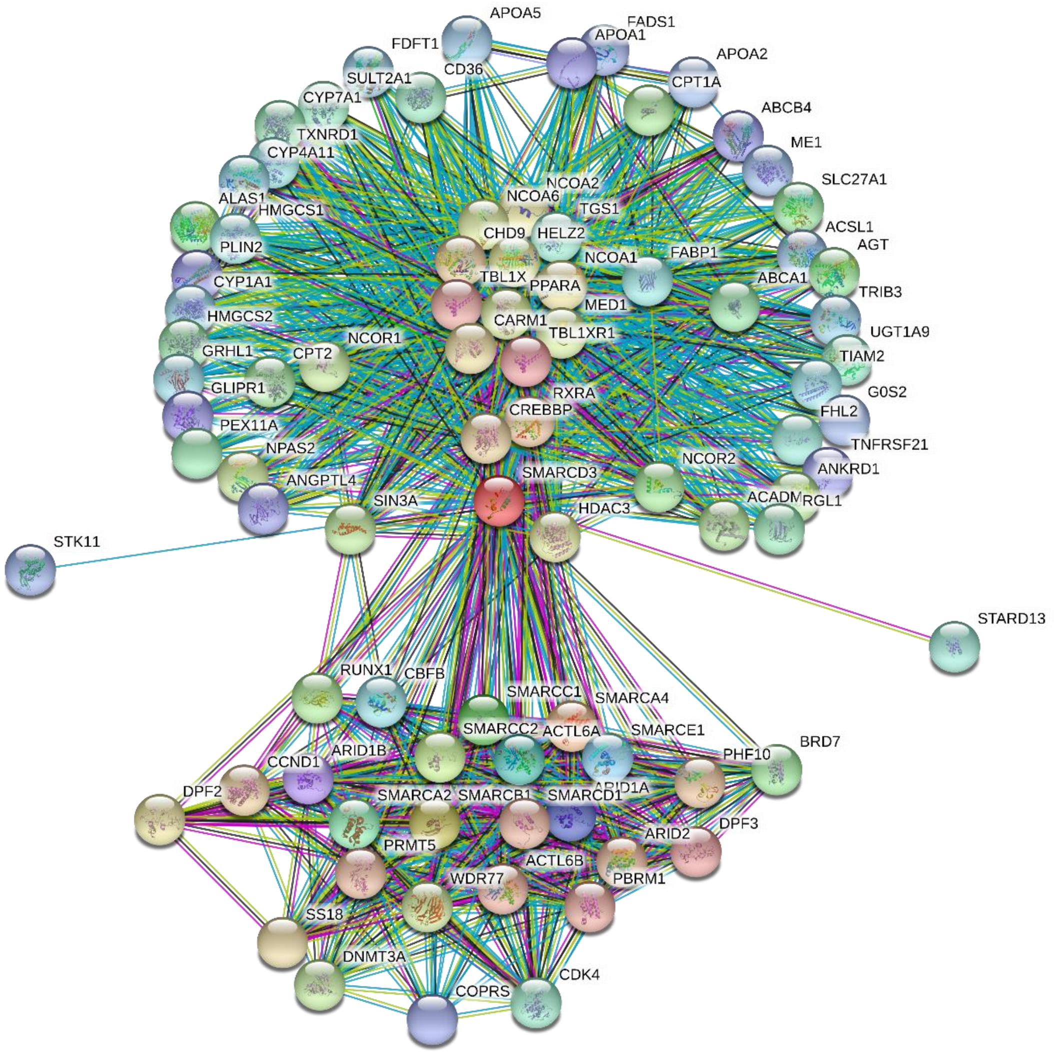

BAF45A/PHF10 (UniProt ID: Q8WUB8; PPID=53.3%; 498 residues)

```
>sp|Q8WUB8|PHF10_HUMAN PHD finger protein 10 OS=Homo sapiens OX=9606 GN=PHF10
PE=1 SV=3
MAAAAGPGAALSPRPCDSDPATPGAQSPKDDNEDNSNDGTQPSKRRRMGSGDSSRSCETSSQDLGFSYYPAENLIEY
KWPPDETGEYYMLQEQVSEYLGVTSFKRKYPDLERRDLSHKEKLYLRELNVITETQCTLGLTALRSDEVIDLMIKEY
PAKHAEYSVILQEKERQRITDHYKEYSQMQQQNTQKVEASKVPEYIKKAAKKAAEFNSNLNRERMEERRAYFDLQTH
VIQVPQGKYKVLPTERTKVSSYPVALIPGQFQEYYKRYSPDELRYLPLNTALYEPPLDPELPALDSDGDSDDGEDGR
GDEKRKNKGTSDSSSGNVSEGESPPDSQEDSFQGRQKSKDKAATPRKDGPKRSVLSKSVPGYKPKVIPNAICGICLK
GKESNKKGKAESLIHCSQCENSGHPSCLDMTMELVSMIKTYPWQCMECKTCIICGQPHHEEEMMFCDMCDRGYHTFC
VGLGAIPSGRWICDCCQRAPPTPRKVGRRGKNSKEG
```

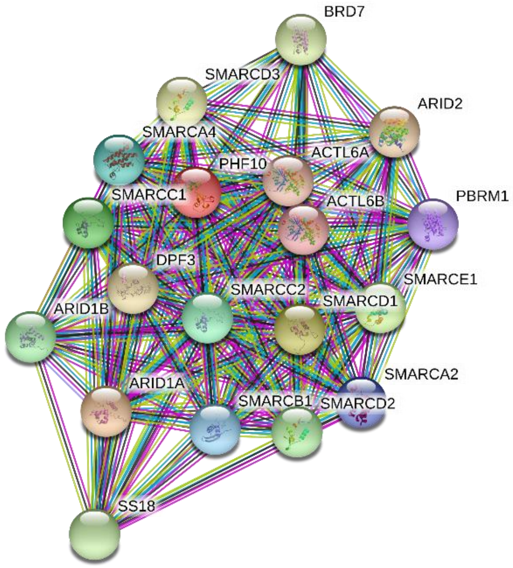

BAF45D/DPF2 (UniProt ID: Q92785; PPID= 59.7%; 391 residues)

>sp|Q92785|REQU_HUMAN Zinc finger protein ubi-d4 OS=Homo sapiens OX=9606 GN=DPF2 PE=1 SV=2
MAAVVENVVKLLGEQYYKDAMEQCHNYNARLCAERSVRLPFLDSQTGVAQSNCYIWMEKRHRGPGLASGQLYSYPAR
RWRKKRRAHPPEDPRLSFPSIKPDTDQTLKKEGLISQDGSSLEALLRTDPLEKRGAPDPRVDDDSLGEFPVTNSRAR
KRILEPDDFLDDLDDEDYEEDTPKRRGKGKSKGKGVGSARKKLDASILEDRDKPYACDICGKRYKNRPGLSYHYAHS
HLAEEEGEDKEDSQPPTPVSQRSEEQKSKKGPDGLALPNNYCDFCLGDSKINKKTGQPEELVSCSDCGRSGHPSCLQ
FTPVMMAAVKTYRWQCIECKCCNICGTSENDDQLLFCDDCDRGYHMYCLTPSMSEPPEGSWSCHLCLDLLKEKASIY
QNQNSS

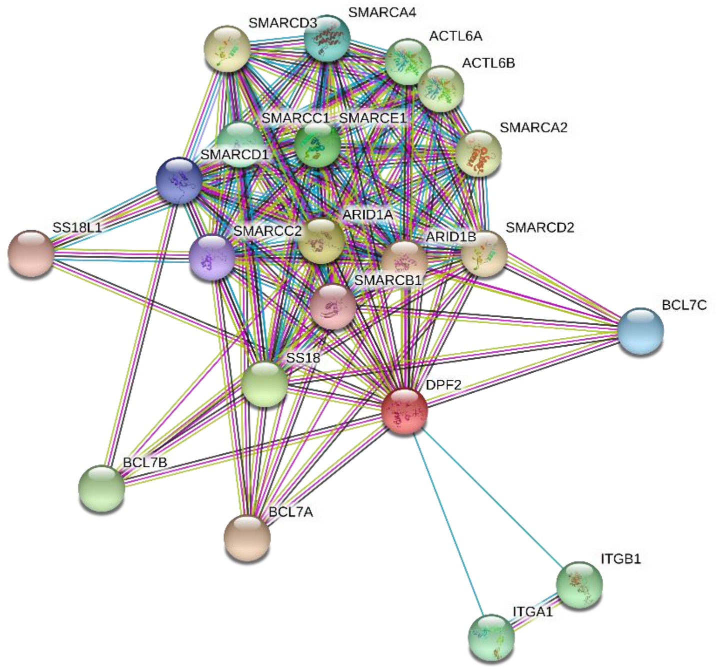

BAF45B/DPF1 (UniProt ID: Q92782; PPID=35.8%; 380 residues)

>sp|Q92782|DPF1_HUMAN Zinc finger protein neuro-d4 OS=Homo sapiens OX=9606 GN=DPF1 PE=1 SV=2
MGGLSARPTAGRTDPAGTCWGQDPGSKMATVIPGPLSLGEDFYREAIEHCRSYNARLCAERSLRLPFLDSQTGVAQN
NCYIWMEKTHRGPGLAPGQIYTYPARCWRKKRRLNILEDPRLRPCEYKIDCEAPLKKEGGLPEGPVLEALLCAETGE
KKIELKEEETIMDCQKQQLLEFPHDLEVEDLEDDIPRRKNRAKGKAYGIGGLRKRQDTASLEDRDKPYVCDKFYKEL
AWVPEAQRKHTAKKAPDGTVIPNGYCDFCLGGSKKTGCPEDLISCADCGRSGHPSCLQFTVNMTAAVRTYRWQCIEC
KSCSLCGTSENDGASWAGLTPQDQLLFCDDCDRGYHMYCLSPPMAEPPEGSWSCHLCLRHLKEKASAYITLT

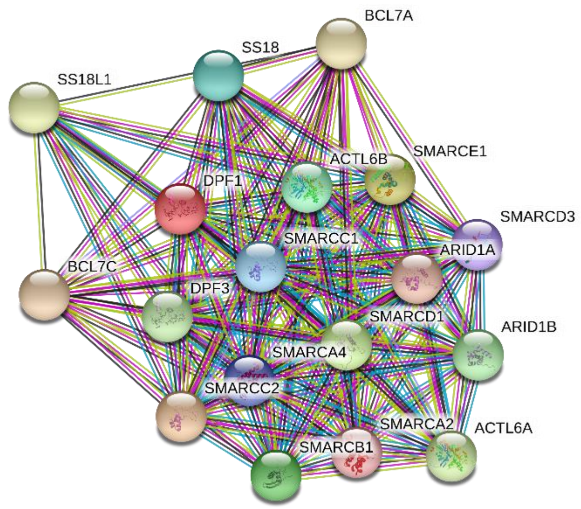

BAF45C/DPF3 (UniProt ID: Q92784; PPID=50.5%; 378 residues)

```
>sp|Q92784|DPF3_HUMAN Zinc finger protein DPF3 OS=Homo sapiens OX=9606
GN=DPF3 PE=1 SV=3
MATVIHNPLKALGDQFYKEAIEHCRSYNSRLCAERSVRLPFLDSQTGVAQNNCYIWMEKRHRGPGLAPGQLYTYPAR
CWRKKRRLHPPEDPKLRLLEIKPEVELPLKKDGFTSESTTLEALLRGEGVEKKVDAREEESIQEIQRVLENDENVEE
GNEEEDLEEDIPKRKNRTRGRARGSAGGRRRHDAASQEDHDKPYVCDICGKRYKNRPGLSYHYAHTHLASEEGDEAQ
DQETRSPPNHRNENHRPQKGPDGTVIPNNYCDFCLGGSNMNKKSGRPEELVSCADCGRSGHPTCLQFTLNMTEAVKT
YKWQCIECKSCILCGTSENDDQLLFCDDCDRGYHMYCLNPPVAEPPEGSWSCHLCWELLKEKASAFGCQA
```

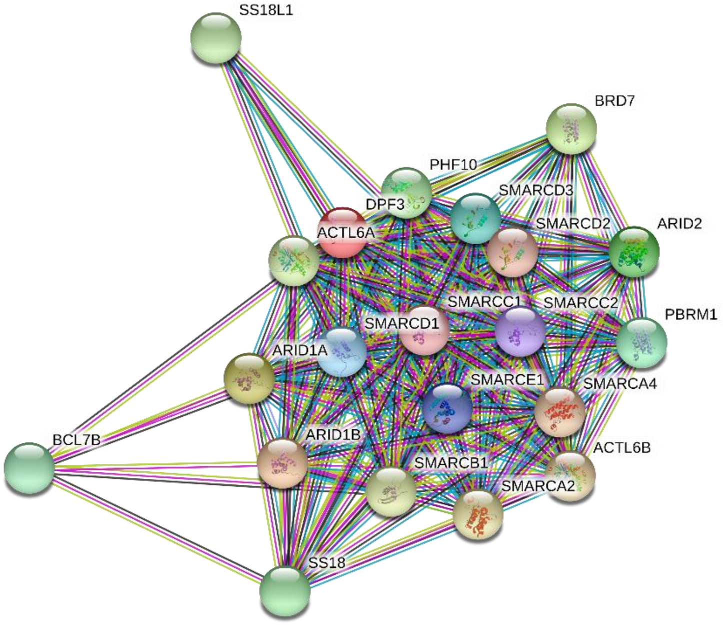

BAF47/SMARCB1 (UniProt ID: Q12824; PPID=35.8%; 385 residues)

```
>sp|Q12824|SNF5_HUMAN SWI/SNF-related matrix-associated actin-dependent
regulator of chromatin subfamily B member 1 OS=Homo sapiens OX=9606
GN=SMARCB1 PE=1 SV=2
MMMMALSKTFGQKPVKFQLEDDGEFYMIGSEVGNYLRMFRGSLYKRYPSLWRRLATVEERKKIVASSHGKKTKPNTK
DHGYTTLATSVTLLKASEVEEILDGNDEKYKAVSISTEPPTYLREQKAKRNSQWVPTLPNSSHHLDAVPCSTTINRN
RMGRDKKRTFPLCFDDHDPAVIHENASQPEVLVPIRLDMEIDGQKLRDAFTWNMNEKLMTPEMFSEILCDDLDLNPL
TFVPAIASAIRQQIESYPTDSILEDQSDQRVIIKLNIHVGNISLVDQFEWDMSEKENSPEKFALKLCSELGLGGEFV
TTIAYSIRGQLSWHQKTYAFSENPLPTVEIAIRNTGDADQWCPLLETLTDAEMEKKIRDQDRNTRRMRRLANTAPAW
```

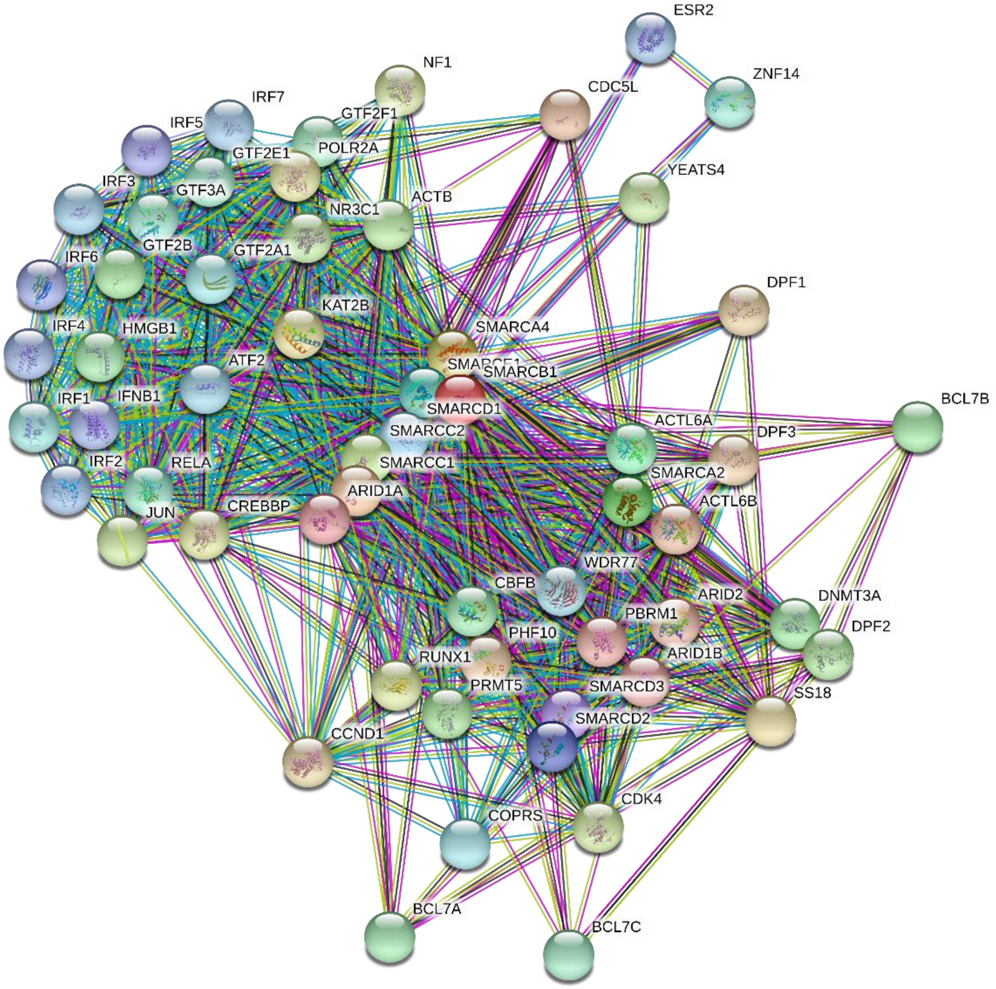

BAF170/SMARCC2 (UniProt ID: Q8TAQ2; PPID=63.1%; 1,214 residues)

```
>sp|Q8TAQ2|SMRC2_HUMAN SWI/SNF complex subunit SMARCC2 OS=Homo sapiens
OX=9606 GN=SMARCC2 PE=1 SV=1
MAVRKKDGGPNVKYYEAADTVTQFDNVRLWLGKNYKKYIQAEPPTNKSLSSLVVQLLQFQEEVFGKHVSNAPLTKLP
IKCFLDFKAGGSLCHILAAAYKFKSDQGWRRYDFQNPSRMDRNVEMFMTIEKSLVQNNCLSRPNIFLCPEIEPKLLG
KLKDIIKRHQGTVTEDKNNASHVVYPVPGNLEEEEWVRPVMKRDKQVLLHWGYYPDSYDTWIPASEIEASVEDAPTP
EKPRKVHAKWILDTDTFNEWMNEEDYEVNDDKNPVSRRKKISAKTLTDEVNSPDSDRRDKKGGNYKKRKRSPSPSPT
PEAKKKNAKKGPSTPYTKSKRGHREEEQEDLTKDMDEPSPVPNVEEVTLPKTVNTKKDSESAPVKGGTMTDLDEQED
ESMETTGKDEDENSTGNKGEQTKNPDLHEDNVTEQTHHIIIPSYAAWFDYNSVHAIERRALPEFFNGKNKSKTPEIY
LAYRNFMIDTYRLNPQEYLTSTACRRNLAGDVCAIMRVHAFLEQWGLINYQVDAESRPTPMGPPPTSHFHVLADTPS
GLVPLQPKTPQQTSASQQMLNFPDKGKEKPTDMQNFGLRTDMYTKKNVPSKSKAAASATREWTEQETLLLLEALEMY
KDDWNKVSEHVGSRTQDECILHFLRLPIEDPYLEDSEASLGPLAYQPIPFSQSGNPVMSTVAFLASVVDPRVASAAA
KSALEEFSKMKEEVPTALVEAHVRKVEEAAKVTGKADPAFGLESSGIAGTTSDEPERIEESGNDEARVEGQATDEKK
EPKEPREGGGAIEEEAKEKTSEAPKKDEEKGKEGDSEKESEKSDGDPIVDPEKEKEPKEGQEEVLKEVVESEGERKT
KVERDIGEGNLSTAAAAALAAAAVKAKHLAAVEERKIKSLVALLVETQMKKLEIKLRHFEELETIMDREREALEYQR
QQLLADRQAFHMEQLKYAEMRARQQHFQQMHQQQQQPPPALPPGSQPIPPTGAAGPPAVHGLAVAPASVVPAPAGSG
APPGSLGPSEQIGQAGSTAGPQQQQPAGAPQPGAVPPGVPPPGPHGPSPFPNQQTPPSMMPGAVPGSGHPGVAGNAP
LGLPFGMPPPPPPPPAPSIIPFGSLADSISINLPAPPNLHGHHHHLPFAPGTLPPPNLPVSMANPLHPNLPATTTMPS
SLPLGPGLGSAAAQSPAIVAAVQGNLLPSASPLPDPGTPLPPDPTAPSPGTVTPVPPPQ
```

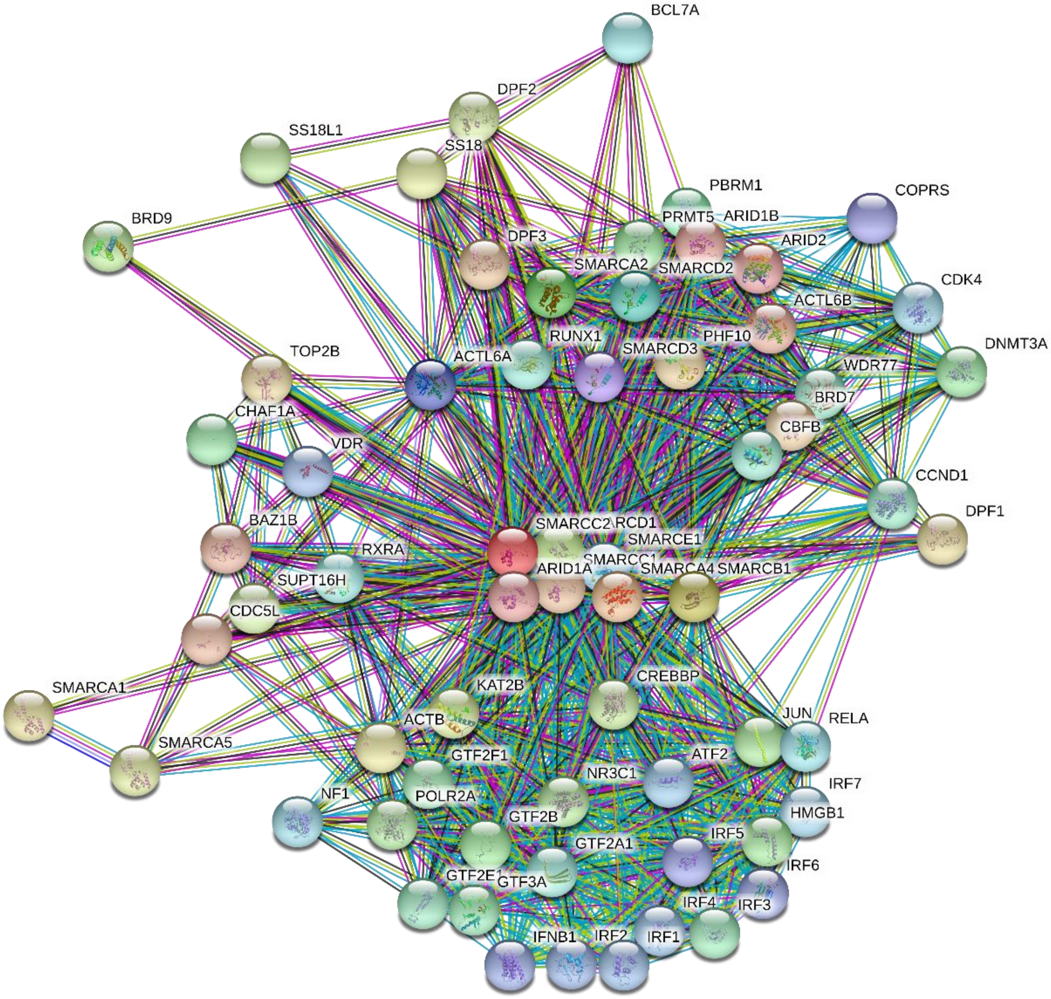

BAF53A/ACTL6A (UniProt ID: O96019; PPID=23.5%; 429 residues)

>sp|O96019|ACL6A_HUMAN Actin-like protein 6A OS=Homo sapiens OX=9606
GN=ACTL6A PE=1 SV=1
MSGGVYGGDEVGALVFDIGSYTVRAGYAGEDCPKVDFPTAIGMVVERDDGSTLMEIDGDKGKQGGPTYYIDTNALRV
PRENMEAISPLKNGMVEDWDSFQAILDHTYKMHVKSEASLHPVLMSEAPWNTRAKREKLTELMFEHYNIPAFFLCKT
AVLTAFANGRSTGLILDSGATHTTAIPVHDGYVLQQGIVKSPLAGDFITMQCRELFQEMNIELVPPYMIASKEAVRE
GSPANWKRKEKLPQVTRSWHNYMCNCVIQDFQASVLQVSDSTYDEQVAAQMPTVHYEFPNGYNCDFGAERLKIPEGL
FDPSNVKGLSGNTMLGVSHVVTTSVGMCDIDIRPGLYGSVIVAGGNTLIQSFTDRLNRELSQKTPPSMRLKLIANNT
TVERRFSSWIGGSILASLGTFQQMWISKQEYEEGGKQCVERKCP

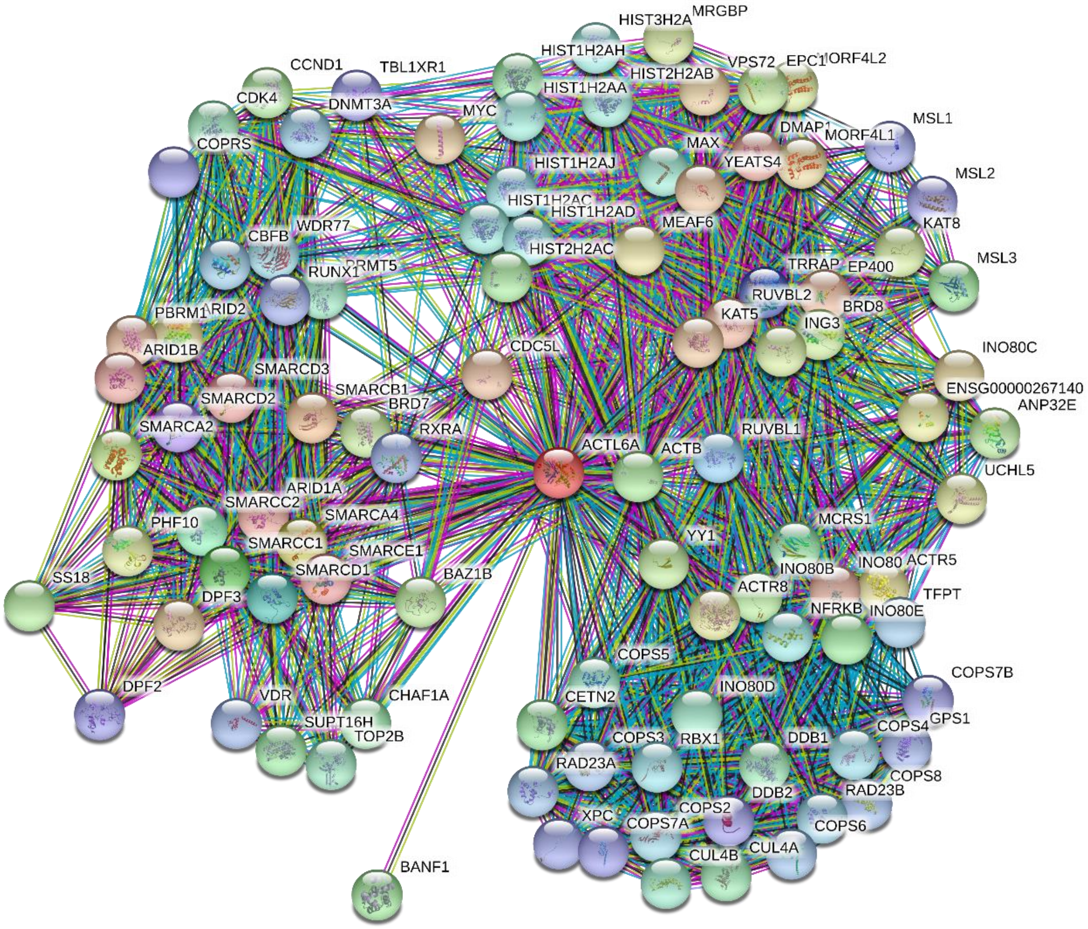

BAF53B/ACTL6B (UniProt ID: O94805; PPID=25.4%; 426 residues)

```
>sp|O94805|ACL6B_HUMAN Actin-like protein 6B OS=Homo sapiens OX=9606
GN=ACTL6B PE=1 SV=1
MSGGVYGGDEVGALVFDIGSFSVRAGYAGEDCPKADFPTTVGLLAAEEGGGLELEGDKEKKGKIFHIDTNALHVPRD
GAEVMSPLKNGMIEDWECFRAILDHTYSKHVKSEPNLHPVLMSEAPWNTRAKREKLTELMFEQYNIPAFFLCKTAVL
TAFANGRSTGLVLDSGATHTTAIPVHDGYVLQQGIVKSPLAGDFISMQCRELFQEMAIDIIPPYMIAAKEPVREGAP
PNWKKKEKLPQVSKSWHNYMCNEVIQDFQASVLQVSDSPYDEQVAAQMPTVHYEMPNGYNTDYGAERLRIPEGLFDP
SNVKGLSGNTMLGVGHVVTTSIGMCDIDIRPGLYGSVIVTGGNTLLQGFTDRLNRELSQKTPPSMRLKLIASNSTME
RKFSPWIGGSILASLGTFQQMWISKQEYEEGGKQCVERKCP
```

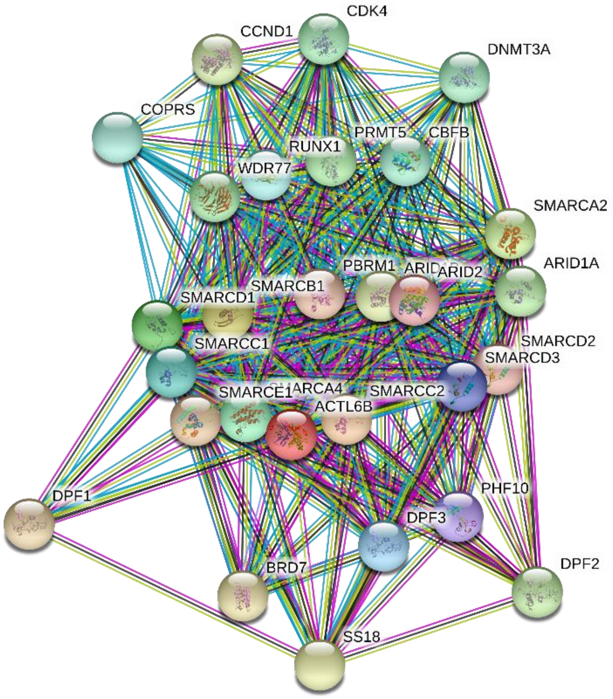

Actin-β/ACTB (UniProt ID: P60709; PPID=22.1%; 375 residues)

```
>sp|P60709|ACTB_HUMAN Actin, cytoplasmic 1 OS=Homo sapiens OX=9606 GN=ACTB
PE=1 SV=1
MDDDIAALVVDNGSGMCKAGFAGDDAPRAVFPSIVGRPRHQGVMVGMGQKDSYVGDEAQSKRGILTLKYPIEHGIVT
NWDDMEKIWHHTFYNELRVAPEEHPVLLTEAPLNPKANREKMTQIMFETFNTPAMYVAIQAVLSLYASGRTTGIVMD
SGDGVTHTVPIYEGYALPHAILRLDLAGRDLTDYLMKILTERGYSFTTTAEREIVRDIKEKLCYVALDFEQEMATAA
SSSSLEKSYELPDGQVITIGNERFRCPEALFQPSFLGMESCGIHETTFNSIMKCDVDIRKDLYANTVLSGGTTMYPG
IADRMQKEITALAPSTMKIKIIAPPERKYSVWIGGSILASLSTFQQMWISKQEYDESGPSIVHRKCF
```

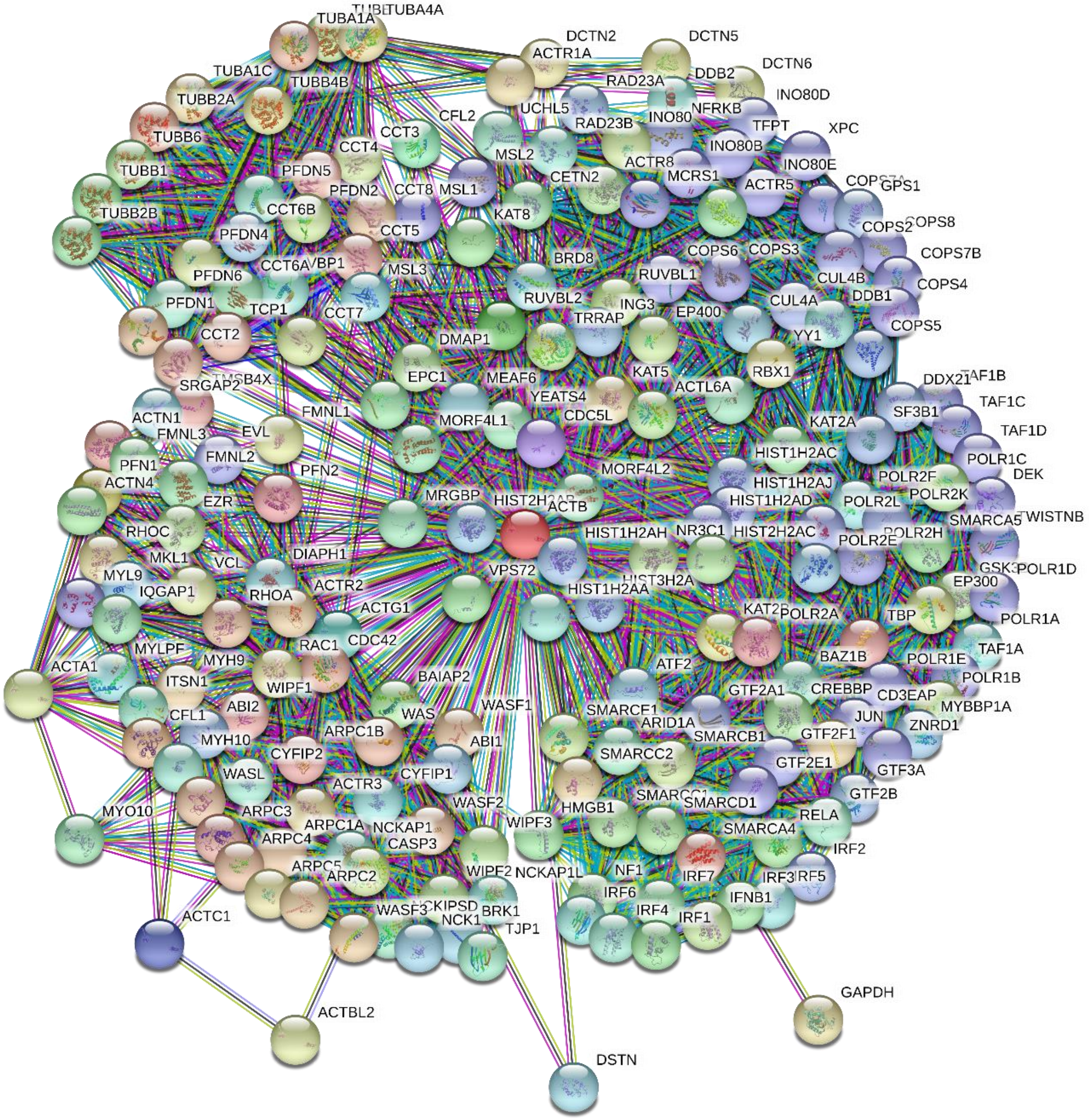

GLTSCR1/BICRA (UniProt ID: Q9NZM4; PPID=88.2%; 1,560 residues)

```
>sp|Q9NZM4|BICRA_HUMAN BRD4-interacting chromatin-remodeling complex-
associated protein OS=Homo sapiens OX=9606 GN=BICRA PE=1 SV=2
MDDEDGRCLLDVICDPQALNDFLHGSEKLDSDDLLDNPGEAQSAFYEGPGLHVQEASGNHLNPEPNQPAPSVDLDFL
EDDILGSPATGGGGGGSGGADQPCDILQQSLQEANITEQTLEAEAELDLGPFQLPTLQPADGGAGPTGAGGAAAVAA
GPQALFPGSTDLLGLQGPPTVLTHQALVPPQDVVNKALSVQPFLQPVGLGNVTLQPIPGLQGLPNGSPGGATAATLG
LAPIQVVGQPVMALNTPTSQLLAKQVPVSGYLASAAGPSEPVTLASAGVSPQGAGLVIQKNLSAAVATTLNGNSVFG
GAGAASAPTGTPSGQPLAVAPGLGSSPLVPAPNVILHRTPTPIQPKPAGVLPPKLYQLTPKPFAPAGATLTIQGEPG
ALPQQPKAPQNLTFMAAGKAGQNVVLSGFPAPALQANVFKQPPATTTGAAPPQPPGALSKPMSVHLLNQGSSIVIPA
QHMLPGQNQFLLPGAPAVQLPQQLSALPANVGGQILAAAAPHTGGQLIANPILTNQNLAGPLSLGPVLAPHSGAHSA
HILSAAPIQVGQPALFQMPVSLAAGSLPTQSQPAPAGPAATTVLQGVTLPPSAVAMLNTPDGLVQPATPAAATGEAA
PVLTVQPAPQAPPAVSTPLPLGLQQPQAQQPPQAPTPQAAAPPQATTPQPSPGLASSPEKIVLGQPPSATPTAILTQ
DSLQMFLPQERSQQPLSAEGPHLSVPASVIVSAPPPAQDPAPATPVAKGAGLGPQAPDSQASPAPAPQIPAAAPLKG
PGPSSSPSLPHQAPLGDSPHLPSPHPTRPPSRPPSRPQSVSRPPSEPPLHPCPPPQAPPTLPGIFVIQNQLGVPPPA
SNPAPTAPGPPQPPLRPQSQPPEGPLPPAPHLPPSSTSSAVASSSETSSRLPAPTPSDFQLQFPPSQGPHKSPTPPP
TLHLVPEPAAPPPPPPRTFQMVTTPFPALPQPKALLERFHQVPSGIILQNKAGGAPAAPQTSTSLGPLTSPAASVLV
SGQAPSGTPTAPSHAPAPAPMAATGLPPLLPAENKAFASNLPTLNVAKAASSGPGKPSGLQYESKLSGLKKPPTLQP
SKEACFLEHLHKHQGSVLHPDYKTAFPSFEDALHRLLPYHVYQGALPSPSDYHKVDEEFETVSTQLLKRTQAMLNKY
RLLLLEESRRVSPSAEMVMIDRMFIQEEKTTLALDKQLAKEKPDEYVSSSRSLGLPIAASSEGHRLPGHGPLSSSAP
GASTQPPPHLPTKLVIRHGGAGGSPSVTWARASSSLSSSSSSSSAASSLDADEDGPMPSRNRPPIKTYEARSRIGLK
LKIKQEAGLSKVVHNTALDPVHQPPPPPATLKVAEPPPRPPPPPPPPTGQMNGTVDHPPPAAPERKPLGTAPHCPRLP
LRKTYRENVGGPGAPEGTPAGRARGGSPAPLPAKVDEATSGLIRELAAVEDELYQRMLKGPPPEPAASAAQGTGDPD
WEAPGLPPAKRRKSESPDVDQASFSSDSPQDDTLTEHLQSAIDSILNLQQAPGRTPAPSYPHAASAGTPASPPPLHR
PEAYPPSSHNGGLGARTLTR
```

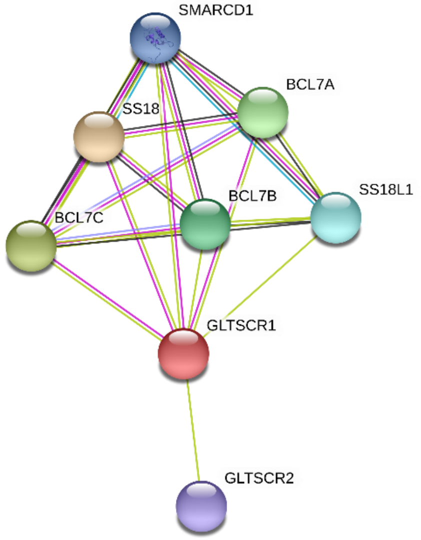

GLTSCR1L/BICRAL (UniProt ID: Q6AI39; PPID=74.8%; 1,079 residues)

```
>sp|Q6AI39|BICRL_HUMAN BRD4-interacting chromatin-remodeling complex-
associated protein-like OS=Homo sapiens OX=9606 GN=BICRAL PE=1 SV=2
MDDDDDSCLLDLIGDPQALNYFLHGPSNKSSNDDLTNAGYSAANSNSIFANSSNADPKSSLKGVSNQLGEGPSDGLP
LSSSLQFLEDELESSPLPDLTEDQPFDILQKSLQEANITEQTLAEEAYLDASIGSSQQFAQAQLHPSSSASFTQASN
VSNYSGQTLQPIGVTHVPVGASFASNTVGVQHGFMQHVGISVPSQHLSNSSQISGSGQIQLIGSFGNHPSMMTINNL
DGSQIILKGSGQQAPSNVSGGLLVHRQTPNGNSLFGNSSSSPVAQPVTVPFNSTNFQTSLPVHNIIIQRGLAPNSNK
VPINIQPKPIQMGQQNTYNVNNLGIQQHHVQQGISFASASSPQGSVVGPHMSVNIVNQQNTRKPVTSQAVSSTGGSI
VIHSPMGQPHAPQSQFLIPTSLSVSSNSVHHVQTINGQLLQTQPSQLISGQVASEHVMLNRNSSNMLRTNQPYTGPM
LNNQNTAVHLVSGQTFAASGSPVIANHASPQLVGGQMPLQQASPTVLHLSPGQSSVSQGRPGFATMPSVTSMSGPSR
FPAVSSASTAHPSLGSAVQSGSSGSNFTGDQLTQPNRTPVPVSVSHRLPVSSSKSTSTFSNTPGTGTQQQFFCQAQK
KCLNQTSPISAPKTTDGLRQAQIPGLLSTTLPGQDSGSKVISASLGTAQPQQEKVVGSSPGHPAVQVESHSGGQKRP
AAKQLTKGAFILQQLQRDQAHTVTPDKSHFRSLSDAVQRLLSYHVCQGSMPTEEDLRKVDNEFETVATQLLKRTQAM
LNKYRCLLLEDAMRINPSAEMVMIDRMFNQEERASLSRDKRLALVDPEGFQADFCCSFKLDKAAHETQFGRSDQHGS
KASSSLQPPAKAQGRDRAKTGVTEPMNHDQFHLVPNHIVVSAEGNISKKTECLGRALKFDKVGLVQYQSTSEEKASR
REPLKASQCSPGPEGHRKTSSRSDHGTESKLSSILADSHLEMTCNNSFQDKSLRNSPKNEVLHTDIMKGSGEPQPDL
QLTKSLETTFKNILELKKAGRQPQSDPTVSGSVELDFPNFSPMASQENCLEKFIPDHSEGVVETDSILEAAVNSILE
C
```

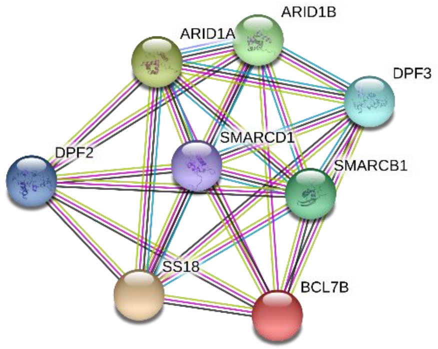

BRM/SMARCA2 (UniProt ID: P51531; PPID=60.6%; 1,590 residues)

```
>sp|P51531|SMCA2_HUMAN Probable global transcription activator SNF2L2 OS=Homo
sapiens OX=9606 GN=SMARCA2 PE=1 SV=2
MSTPTDPGAMPHPGPSPGPGPSPGPILGPSPGPGPSPGSVHSMMGPSPGPPSVSHPMPTMGSTDFPQEGMHQMHKPI
DGIHDKGIVEDIHCGSMKGTGMRPPHPGMGPPQSPMDQHSQGYMSPHPSPLGAPEHVSSPMSGGGPTPPQMPPSQPG
ALIPGDPQAMSQPNRGPSPFSPVQLHQLRAQILAYKMLARGQPLPETLQLAVQGKRTLPGLQQQQQQQQQQQQQQQQ
QQQQQQQPQQQPPQPQTQQQQPALVNYNRPSGPGPELSGPSTPQKLPVPAPGGRPSPAPPAAAQPPAAAVPGPSVP
QPAPGQPSPVLQLQQKQSRISPIQKPQGLDPVEILQEREYRLQARIAHRIQELENLPGSLPPDLRTKATVELKALRL
LNFQRQLRQEVVACMRRDTTLETALNSKAYKRSKRQTLREARMTEKLEKQQKIEQERKRRQKHQEYLNSILQHAKDF
KEYHRSVAGKIQKLSKAVATWHANTEREQKKETERIEKERMRRLMAEDEEGYRKLIDQKKDRRLAYLLQQTDEYVAN
LTNLVWEHKQAQAAKEKKKRRRRKKKAEENAEGGESALGPDGEPIDESSQMSDLPVKVTHTETGKVLFGPEAPKASQ
LDAWLEMNPGYEVAPRSDSEESDSDYEEEDEEEESSRQETEEKILLDPNSEEVSEKDAKQIIETAKQDVDDEYSMQY
SARGSQSYYTVAHAISERVEKQSALLINGTLKHYQLQGLEWMVSLYNNNLNGILADEMGLGKTIQTIALITYLMEHK
RLNGPYLIIVPLSTLSNWTYEFDKWAPSVVKISYKGTPAMRRSLVPQLRSGKFNVLLTTYEYIIKDKHILAKIRWKY
MIVDEGHRMKNHHCKLTQVLNTHYVAPRRILLTGTPLQNKLPELWALLNFLLPTIFKSCSTFEQWFNAPFAMTGERV
DLNEEETILIIRRLHKVLRPFLLRRLKKEVESQLPEKVEYVIKCDMSALQKILYRHMQAKGILLTDGSEKDKKGKGG
AKTLMNTIMQLRKICNHPYMFQHIEESFAEHLGYSNGVINGAELYRASGKFELLDRILPKLRATNHRVLLFCQMTSL
MTIMEDYFAFRNFLYLRLDGTTKSEDRAALLKKFNEPGSQYFIFLLSTRAGGLGLNLQAADTVVIFDSDWNPHQDLQ
AQDRAHRIGQQNEVRVLRLCTVNSVEEKILAAAKYKLNVDQKVIQAGMFDQKSSSHERRAFLQAILEHEEENEEEDE
VPDDETLNQMIARREEEFDLFMRMDMDRRREDARNPKRKPRLMEEDELPSWIIKDDAEVERLTCEEEEEKIFGRGSR
QRRDVDYSDALTEKQWLRAIEDGNLEEMEEEVRLKKRKRRRNVDKDPAKEDVEKAKKRRGRPPAEKLSPNPPKLTKQ
MNAIIDTVINYKDRCNVEKVPSNSQLEIEGNSSGRQLSEVFIQLPSRKELPEYYELIRKPVDFKKIKERIRNHKYRS
LGDLEKDVMLLCHNAQTFNLEGSQIYEDSIVLQSVFKSARQKIAKEEESEDESNEEEEEEDEEESESEAKSVKVKIK
LNKKDDKGRDKGKGKKRPNRGKAKPVVSDFDSDEEQDEREQSEGSGTDDE
```

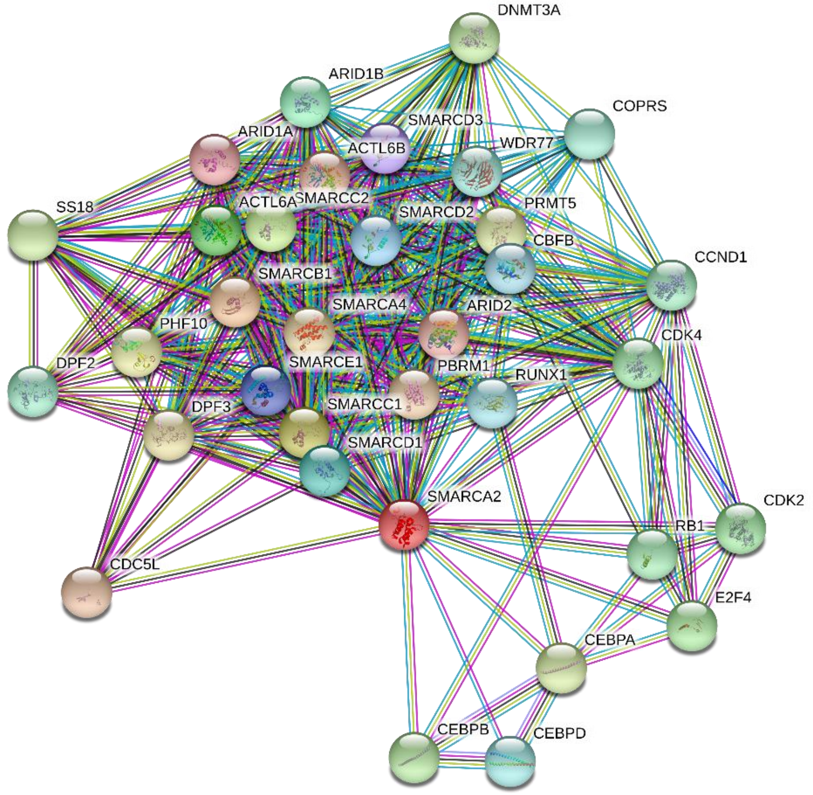

Supplement: Supplementary file 1 [file ijms-20-05260-s001.pdf]
